# Combined exercise for body composition and cardiometabolic health in obese young people meta analysis

**DOI:** 10.1016/j.isci.2026.116888

**Published:** 2026-07-21

**Authors:** Jingyou Zhong, Yanhao Wang, Hongliang Wu, Huangkun Chen, Ming Li

**Affiliations:** 1 School of Physical Education and Sport Science, Fujian Normal University, Fuzhou, China

**Keywords:** combined exercise, aerobic exercise, obesity, body composition, cardiometabolic risk, meta-analysis, adolescents, young adults

## Abstract

Combined exercise (CE) may offer benefits beyond aerobic exercise (AE) alone for individuals with overweight or obesity, but comparative evidence remains uncertain. We performed a systematic review and meta-analysis of randomized trials comparing CE with AE in adolescents and young adults aged 6–45 years. Twenty-nine studies involving 1,383 participants were included. Compared with AE, CE was associated with greater improvements in lean body mass and insulin-resistance indicators, including insulin and HOMA-IR. No consistent between-group differences were observed for body weight, BMI, body fat percentage, waist circumference, systolic blood pressure, or lipid outcomes, while HDL-C showed a small difference favoring AE. Secondary and exploratory analyses should be interpreted cautiously because several outcomes were supported by limited studies and low-to-very-low certainty evidence. These findings suggest that CE may be useful for selected body composition and metabolic outcomes, but does not appear universally superior to AE.

## Highlights

• Combined exercise improved lean body mass versus aerobic exercise
• Insulin and HOMA-IR favored combined exercise in pooled analyses
• Adiposity, blood pressure, and lipid outcomes showed inconsistent effects
• Evidence certainty was mostly low to very low across outcomes

## Graphical abstract

**Figure:**
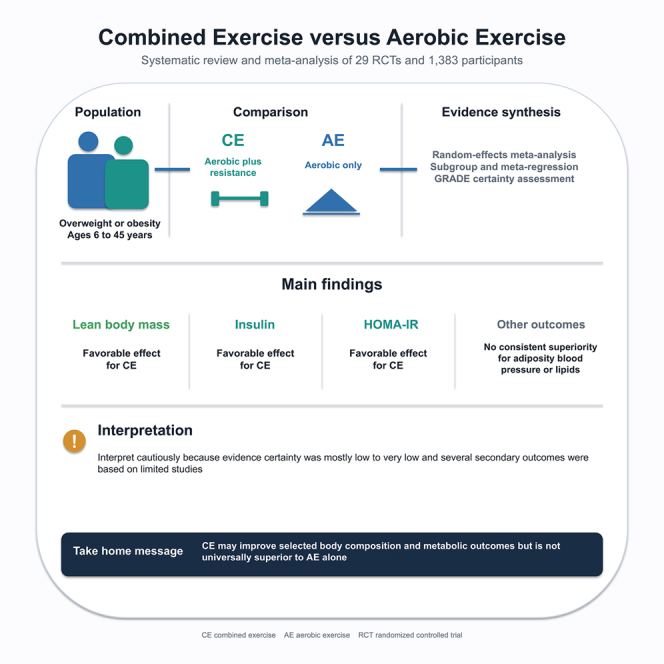

## Teaser

Health sciences; Medicine; Medical specialty; Obesity medicine; Human activity in medical context; Physical activity; Procedure; Obesity prevention in child health

## Introduction

In recent years, overweight and obesity have emerged as formidable global public health challenges confronting adolescent and young adult populations. Their sustained proliferation not only profoundly compromises individual biopsychosocial development but also substantially heightens the risk of multifaceted chronic diseases in adulthood.^1^^,^^2^ Obesity, particularly central adiposity, is intimately linked to cardiometabolic risks within this demographic, typically manifesting as insulin resistance, dyslipidemia, endothelial dysfunction, and early-onset cardiovascular disease.^3^^,^^4^ Empirical evidence suggests that excessive adiposity during adolescence triggers a persistent state of systemic low-grade inflammation, which directly impairs cardiovascular resilience and metabolic health.^5^^,^^6^ Furthermore, the deleterious alterations in body composition associated with obesity—characterized by elevated body fat percentage (BFP) and a relative deficit in lean body mass (LBM)—are particularly pronounced during the critical transition from adolescence to adulthood, further exacerbating the incidence of metabolic syndrome.^7^^,^^8^ Consequently, exploring efficacious and sustainable lifestyle intervention strategies to optimize body composition and mitigate cardiometabolic risk in this high-risk cohort has become a pivotal imperative in contemporary clinical medicine and public health.^9^^,^^10^

Regular exercise interventions, serving as non-invasive and cost-effective non-pharmacological modalities, have been broadly substantiated as foundational therapies for ameliorating body composition and cardiometabolic health in adolescents and young adults with obesity.^11^^,^^12^ Conventional aerobic exercise (AE) has been associated with reductions in body weight (BW) and adiposity and may improve cardiorespiratory fitness, circulating insulin levels, and lipid metabolism.^13^^,^^14^^,^^15^ Nevertheless, isolated AE presents inherent limitations regarding the augmentation of fat-free mass and muscular fitness. Conversely, resistance training (RT)—an equally critical modality—not only stimulates skeletal muscle hypertrophy and elevates the basal metabolic rate, but also significantly bolsters insulin sensitivity, independent of weight reduction, by facilitating skeletal muscle glucose uptake.^16^^,^^17^^,^^18^^,^^19^ Given the physiological and mechanistic complementarity of these distinct exercise modalities, combined exercise (CE) is theoretically postulated to engender synergistic adaptations. Consequently, CE may have potential to improve body composition and selected cardiometabolic parameters in populations with obesity, although its added value over AE alone may vary across outcomes.^20^^,^^21^

Recent evidence further supports the clinical relevance of different exercise modalities for individuals with excess BW. A network meta-analysis of 81 randomized controlled trials showed that AE, RT, CE, high-intensity interval training (HIIT), and hybrid-type exercise interventions can produce favorable effects on cardiometabolic outcomes in adults with overweight or obesity, although the magnitude of benefit may vary across exercise modalities and outcome domains.^22^ In addition, HIIT has been proposed as a feasible and time-efficient strategy for improving physical fitness, metabolic health, and cardiovascular function in populations with impaired metabolic health.^23^ RT has also been shown to improve several cardiometabolic health-related indices in patients with type 2 diabetes mellitus (T2DM) and overweight or obesity, including markers of body composition, lipid metabolism, and glycemic control.^24^ Moreover, CE has gained increasing attention because it integrates the metabolic and cardiorespiratory benefits of AE with the muscle-strengthening effects of RT, and recent systematic evidence suggests that it may be particularly useful for improving body composition when compared with single-mode exercise programs.^25^ The growing popularity of personalized, evidence-based, and hybrid exercise programming in contemporary fitness practice further underscores the practical importance of identifying more suitable exercise modalities for specific populations and outcomes.^26^ However, most previous meta-analyses have evaluated exercise modalities against non-exercise control conditions or compared multiple exercise types within broader network frameworks, rather than directly quantifying the incremental value of adding RT to an AE program.

Several recent randomized trials have directly compared CE with AE or examined exercise dose-response effects in populations with obesity.^27^^,^^28^ Although prior meta-analyses have examined the effects of different exercise modalities in populations with overweight or obesity,^29^^,^^30^^,^^31^ several important gaps remain. Most available syntheses have focused on exercise versus non-exercise controls, single-mode exercise interventions, or broad network comparisons, leaving uncertainty regarding the direct comparative effectiveness of CE versus AE alone. This distinction is clinically important because AE remains one of the most commonly prescribed exercise modalities for obesity management, whereas CE requires additional resistance-training components, equipment, supervision, and progression planning. Therefore, determining whether CE provides benefits beyond AE alone has direct implications for exercise prescription. In addition, previous reviews have often focused on either pediatric or adult populations separately,^29^^,^^30^ whereas obesity-related metabolic risk commonly emerges during adolescence and tracks into adulthood. We therefore included adolescents and adults to capture this developmental continuum and to improve the applicability of findings across transitional and adult age groups. Nevertheless, we recognize that age, developmental stage, baseline disease status, and intervention characteristics may influence metabolic and training adaptations.^32^^,^^33^ Accordingly, age group, baseline BMI, intervention duration, training frequency, training sequence, and exercise intensity were examined as potential moderators, and subgroup and meta-regression findings were interpreted cautiously as exploratory analyses. Based on these gaps, the present systematic review and meta-analysis aimed to directly compare CE with AE alone for body composition and cardiometabolic risk outcomes in adolescents and adults with overweight or obesity.

## Results

### Literature search and selection results

**Figure 1:**
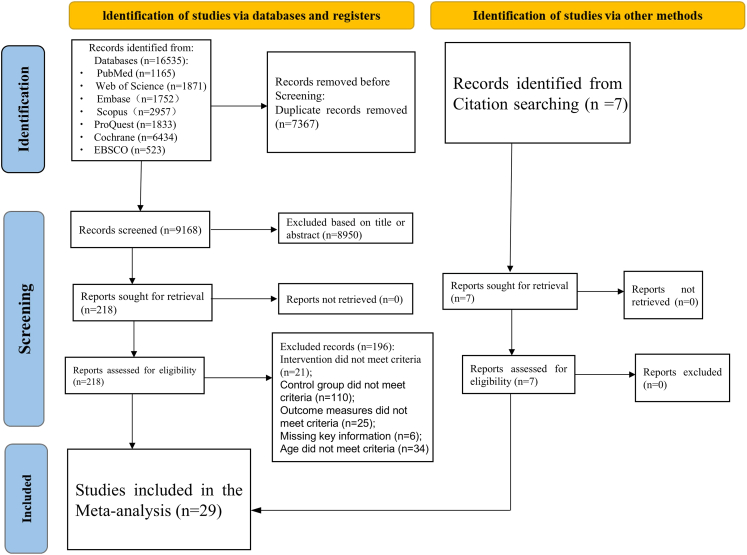
Literature screening flowchart The flowchart summarizes the identification, screening, eligibility assessment, and final inclusion process for this systematic review and meta-analysis. A total of 16,542 records were identified through database searching, and 7 additional records were identified through citation searching. After duplicate removal, titles and abstracts were screened, followed by full-text eligibility assessment. Studies were excluded at the full-text stage mainly because of ineligible control groups, inappropriate participant age, lack of relevant outcome measures, ineligible intervention protocols, or missing critical data. Ultimately, 29 randomized controlled trials were included in the systematic review and meta-analysis. Abbreviations: PRISMA, Preferred Reporting Items for Systematic Reviews and Meta-Analyses; RCT, randomized controlled trial.

The initial comprehensive database search yielded a total of 16,542 bibliographic records. Following the removal of 7,367 duplicate records, an initial screening of titles and abstracts excluded 8,950 demonstrably irrelevant articles. Subsequently, the remaining 225 articles underwent rigorous full-text evaluation for eligibility. Of these, 196 reports were further excluded for the following predetermined reasons: ineligible control group (*n* = 110), inappropriate participant age (*n* = 34), lack of relevant outcome measures (*n* = 25), ineligible intervention protocol (*n* = 21), and missing critical data (*n* = 6). Ultimately, 29 independent studies^34^^,^^35^^,^^36^^,^^37^^,^^38^^,^^39^^,^^40^^,^^41^^,^^42^^,^^43^^,^^44^^,^^45^^,^^46^^,^^47^^,^^48^^,^^49^^,^^50^^,^^51^^,^^52^^,^^53^^,^^54^^,^^55^^,^^56^^,^^57^^,^^58^^,^^59^^,^^60^^,^^61^^,^^62^ completely satisfied the stringent inclusion criteria and were incorporated into the present systematic review and meta-analysis. The detailed study selection process is delineated in the PRISMA flowchart (Figure 1).

### Baseline characteristics of the included studies

This study ultimately incorporated 29 independent RCTs. The baseline mean age of the participants ranged from 10.05 to 45.64 years, with baseline mean BMI values ranging from 25.3 to 39.7 kg/m^2^. The demographic profile predominantly comprised individuals with uncomplicated overweight or obesity; however, select cohorts concurrently presented with metabolic aberrations, including hypertension, T2DM, or metabolic syndrome. Regarding the design of the exercise intervention protocols, the intervention duration across the included trials exhibited moderate heterogeneity, spanning from a brief 4 weeks to an extended 52 weeks. The prescribed training frequency was predominantly concentrated at 3 to 6 sessions per week, with the duration of individual exercise sessions largely controlled between 45 and 67 min. CE was primarily operationalized by alternating or superimposing AE and RT, either within the same session or on alternating days. Notably, the overwhelming majority of studies maintained strict isocaloric or time-matched conditions between the CE and isolated AE protocols. Furthermore, the included trials demonstrated high methodological quality concerning outcome assessments; body composition was rigorously evaluated utilizing objective and extensively validated measurement modalities, such as dual-energy X-ray absorptiometry, magnetic resonance imaging, air displacement plethysmography, or bioelectrical impedance analysis. A comprehensive delineation of the baseline study characteristics and the specific exercise intervention parameters is provided in Tables S1 and S2.

### Risk of bias assessment results

**Figure 2:**
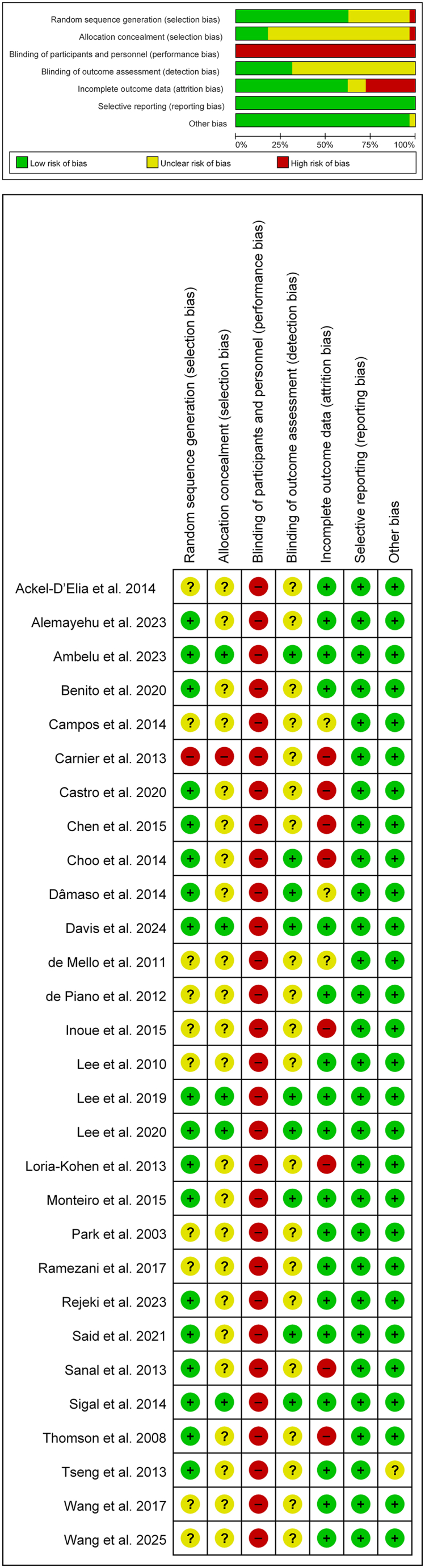
Risk of bias assessment of included studies The figure presents the risk of bias assessment for the 29 included randomized controlled trials across the Cochrane risk-of-bias domains, including random sequence generation, allocation concealment, blinding of participants and personnel, blinding of outcome assessment, incomplete outcome data, selective reporting, and other bias. Green symbols indicate low risk of bias, yellow symbols indicate unclear risk of bias or some concerns, and red symbols indicate high risk of bias. Because of the nature of exercise interventions, all included trials were judged as having high risk of bias for blinding of participants and personnel. In contrast, selective reporting was generally assessed as low risk across studies. Several studies had unclear risk in allocation concealment and blinding of outcome assessment because methodological details were insufficiently reported. Abbreviations: RCT, randomized controlled trial; RoB, risk of bias.

The methodological quality of the 29 included trials was appraised utilizing the Cochrane risk-of-bias tool. Constrained by the intrinsic nature of exercise interventions, all included trials inevitably exhibited a high risk of bias concerning the blinding of participants and personnel. Conversely, all studies demonstrated a universally low risk regarding the selection of the reported results. The predominant majority of the trials employed adequate random sequence generation and maintained the integrity of their outcome data. However, within the domains of allocation concealment and blinding of outcome assessment, a substantial proportion of the studies were categorized as having “some concerns” due to the insufficient reporting of specific methodological details. Overall, the methodological quality of the included studies was deemed moderately acceptable. A comprehensive delineation of the risk of bias assessment is illustrated in Figure 2.

### Meta-analysis results

#### BW

**Figure 3:**
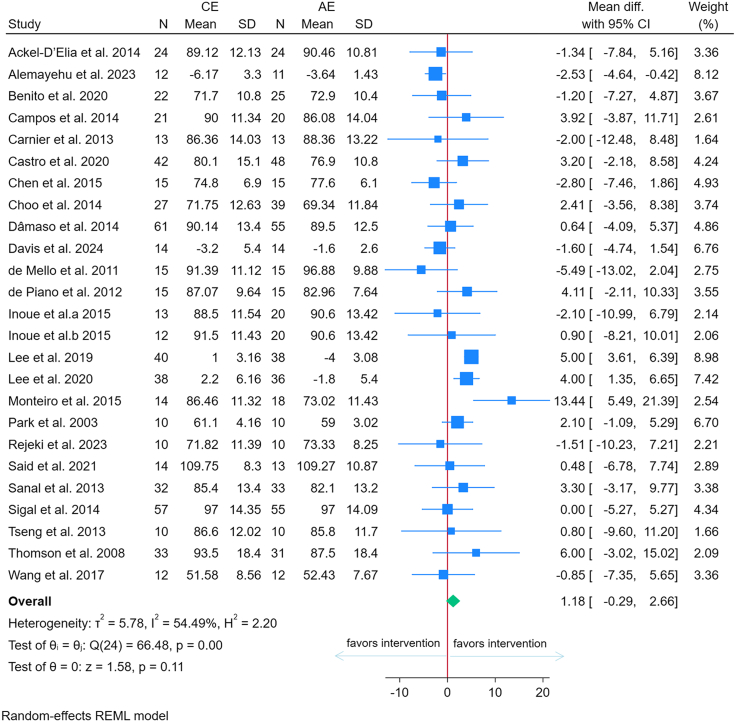
Meta-analysis results for body weight The forest plot shows the pooled effect of combined exercise compared with aerobic exercise on post-intervention body weight in adolescents and adults with overweight or obesity. The analysis included 25 independent studies. The pooled mean difference was 1.18 kg, with a 95% confidence interval from −0.29 to 2.66, indicating no statistically significant between-group difference between combined exercise and aerobic exercise. Between-study heterogeneity was moderate, with τ^2^ = 5.78, I^2^ = 54.49%, and H^2^ = 2.20. Each square represents the effect estimate from an individual study, with square size proportional to study weight. Horizontal lines represent 95% confidence intervals, and the diamond represents the pooled random-effects estimate. Negative values indicate a greater reduction in body weight in the combined exercise group, whereas positive values indicate a greater reduction in the aerobic exercise group. Abbreviations: CE, combined exercise; AE, aerobic exercise; BW, body weight; MD, mean difference; CI, confidence interval; SD, standard deviation; REML, restricted maximum likelihood; τ^2^, between-study variance; I^2^, proportion of total variability due to between-study heterogeneity; H^2^, relative excess in Q statistic over its degrees of freedom; Q, Cochran’s heterogeneity statistic.

The meta-analysis, incorporating 25 independent studies, showed no statistically significant between-group difference between CE and AE in post-intervention BW among adolescents and adults with overweight or obesity (MD = 1.18 kg, 95% CI: −0.29 to 2.66, *p* = 0.11). Between-study heterogeneity was moderate (τ^2^ = 5.78, I^2^ = 54.49%, H^2^ = 2.20; Q(24) = 66.48, *p* < 0.001) (Figure 3).

Exploratory subgroup analysis for BW (Table S3) suggested that CE was associated with greater BW reduction in interventions lasting ≤12 weeks, whereas AE appeared more favorable in interventions lasting >12 weeks. Regarding training sequence, CE protocols using an aerobic-prior-to-resistance paradigm appeared to show less favorable weight-loss effects than isolated AE. Furthermore, no significant subgroup effects were detected concerning age, BMI, training frequency, average training duration per session, total weekly training duration, or exercise intensity (*p* > 0.05).

Meta-regression analyses were conducted to evaluate the potential moderating effects of the following continuous variables on the pooled main effect: age (*β* = −0.08, 95% *CI*: −0.20 to 0.04, *p* = 0.20), baseline BMI (*β* = 0.03, 95% *CI*: −0.36 to 0.43, *p* = 0.88), intervention duration (*β* = 0.02, 95% *CI*: −0.08 to 0.12, *p* = 0.67), training frequency (*β* = 0.30, 95% *CI*: −1.45 to 2.06, *p* = 0.74), average training duration per session (*β* = 0.27, 95% *CI*: −0.31 to 0.85, *p* = 0.35), and total weekly training duration (*β* = 0.00, 95% *CI*: −0.03 to 0.04, *p* = 0.76). Meta-regression did not identify statistically significant associations between the examined continuous variables and the comparative BW effect of CE versus AE (*p* > 0.05).

#### BMI

**Figure 4:**
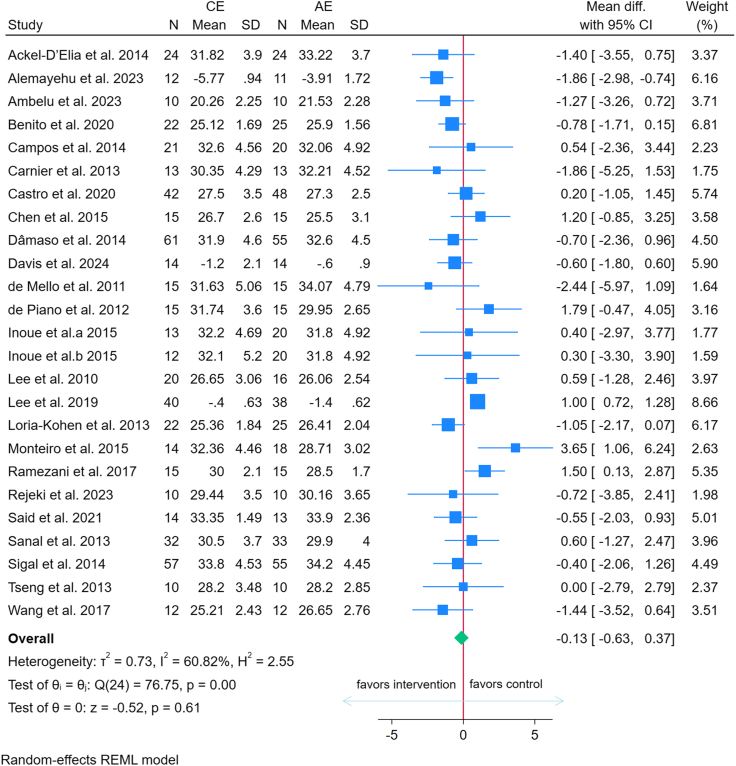
Meta-analysis results for body mass index The forest plot shows the pooled effect of combined exercise compared with aerobic exercise on post-intervention body mass index. The analysis included 25 independent studies and showed no statistically significant between-group difference between the two exercise modalities. The pooled mean difference was −0.13 kg/m^2^, with a 95% confidence interval from −0.63 to 0.37. Between-study heterogeneity was moderate to substantial, with τ^2^ = 0.73, I^2^ = 60.82%, and H^2^ = 2.55. Each square represents the effect estimate from an individual study, with square size proportional to study weight. Horizontal lines represent 95% confidence intervals, and the diamond represents the pooled random-effects estimate. Negative values indicate a greater reduction in body mass index in the combined exercise group, whereas positive values indicate a greater reduction in the aerobic exercise group. Abbreviations: CE, combined exercise; AE, aerobic exercise; BMI, body mass index; MD, mean difference; CI, confidence interval; SD, standard deviation; REML, restricted maximum likelihood; τ^2^, between-study variance; I^2^, proportion of total variability due to between-study heterogeneity; H^2^, relative excess in Q statistic over its degrees of freedom; Q, Cochran’s heterogeneity statistic.

The meta-analysis, incorporating 25 independent studies, showed no statistically significant between-group difference between CE and AE in post-intervention BMI among adolescents and adults with overweight or obesity (MD = −0.13 kg/m^2^, 95% CI: −0.63 to 0.37, *p* = 0.61). Between-study heterogeneity was moderate to substantial (τ^2^ = 0.73, I^2^ = 60.82%, H^2^ = 2.55; Q(24) = 76.75, *p* < 0.001) (Figure 4).

Subgroup analysis for BMI (Table S4) revealed that within the cohorts of adults, individuals with overweight, and protocols employing moderate-intensity interventions, the efficacy of CE in reducing BMI was significantly superior to that of isolated AE. Furthermore, no significant subgroup effects were observed concerning intervention duration, training frequency, average training duration per session, total weekly training duration, or training sequence (*p* > 0.05).

**Figure 5:**
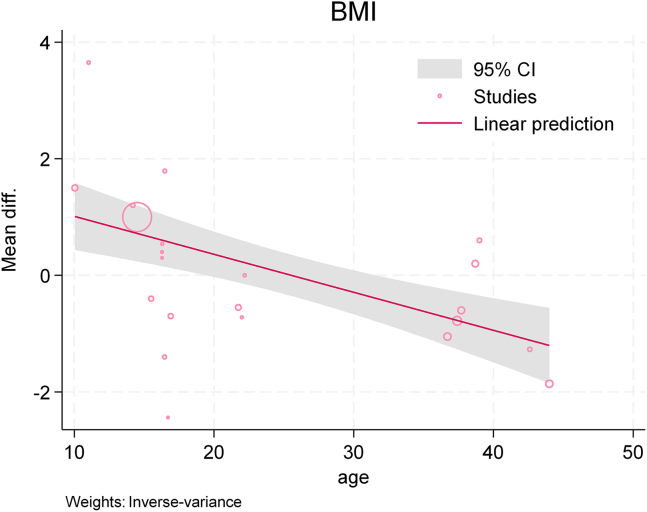
Regression analysis results for body mass index The meta-regression plot shows the association between participant age and the comparative effect of combined exercise versus aerobic exercise on body mass index. The fitted regression line suggests a possible age-related pattern, with combined exercise appearing more favorable for body mass index reduction in older participants. The meta-regression coefficient for age was β = −0.07, with a 95% confidence interval from −0.09 to −0.04, suggesting a statistically significant study-level association. Each circle represents an individual study, with circle size reflecting inverse-variance weight. The solid line represents the linear prediction, and the shaded area represents the 95% confidence interval. Because this was an exploratory study-level meta-regression, the finding should be interpreted cautiously. Abbreviations: CE, combined exercise; AE, aerobic exercise; BMI, body mass index; CI, confidence interval; β, meta-regression coefficient.

Meta-regression analyses were conducted to evaluate the potential moderating effects of the following continuous variables: age (*β* = −0.07, 95% *CI*: −0.09 to −0.04, *p* < 0.01), baseline BMI (*β* = 0.04, 95% *CI*: −0.09 to 0.17, *p* = 0.57), intervention duration (*β* = 0.00, 95% *CI*: −0.04 to 0.03, *p* = 0.94), training frequency (*β* = 0.22, 95% *CI*: −0.86 to 1.30, *p* = 0.69), average training duration per session (*β* = −0.01, 95% *CI*: −0.18 to 0.15, *p* = 0.87), and total weekly training duration (*β* = 0.00, 95% *CI*: −0.01 to 0.02, *p* = 0.66). Meta-regression suggested a possible age-related pattern in the comparative BMI effect of CE versus AE (*p* < 0.01), with CE appearing more favorable in older participants (Figure 5). Conversely, baseline BMI, intervention duration, training frequency, average training duration per session, and total weekly training duration did not show significant moderating effects (*p* > 0.05).

#### BFP

**Figure 6:**
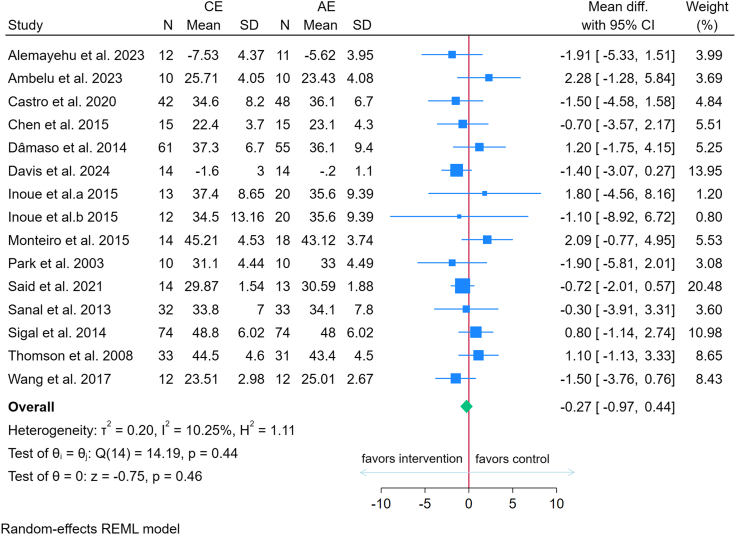
Meta-analysis results for body fat percentage The forest plot shows the pooled effect of combined exercise compared with aerobic exercise on post-intervention body fat percentage. The analysis included 15 independent studies and showed no statistically significant between-group difference. The pooled mean difference was −0.27%, with a 95% confidence interval from −0.97 to 0.44. Between-study heterogeneity was low and not statistically significant, with τ^2^ = 0.20, I^2^ = 10.25%, and H^2^ = 1.11. Each square represents the effect estimate from an individual study, with square size proportional to study weight. Horizontal lines represent 95% confidence intervals, and the diamond represents the pooled random-effects estimate. Negative values indicate a greater reduction in body fat percentage in the combined exercise group, whereas positive values indicate a greater reduction in the aerobic exercise group. Abbreviations: CE, combined exercise; AE, aerobic exercise; BFP, body fat percentage; MD, mean difference; CI, confidence interval; SD, standard deviation; REML, restricted maximum likelihood; τ^2^, between-study variance; I^2^, proportion of total variability due to between-study heterogeneity; H^2^, relative excess in Q statistic over its degrees of freedom; Q, Cochran’s heterogeneity statistic.

The meta-analysis, incorporating 15 independent studies, showed no statistically significant between-group difference between CE and AE in post-intervention BFP among adolescents and adults with overweight or obesity (MD = −0.27%, 95% CI: −0.97 to 0.44, *p* = 0.46). Between-study heterogeneity was low and not statistically significant (τ^2^ = 0.20, I^2^ = 10.25%, H^2^ = 1.11; Q(14) = 14.19, *p* = 0.44) (Figure 6).

Subgroup analysis for BFP (Table S5) revealed that for protocols employing moderate-intensity interventions, the efficacy of CE in reducing BFP was significantly superior to that of isolated AE. Furthermore, no significant subgroup effects were observed concerning baseline BMI, intervention duration, training frequency, average training duration per session, total weekly training duration, or training sequence (*p* > 0.05).

Meta-regression analyses were conducted to evaluate the potential moderating effects of the following continuous variables on the pooled main effect: age (*β* = −0.06, 95% *CI*: −0.12 to 0.01, *p* = 0.09), baseline BMI (*β* = 0.06, 95% *CI*: −0.14 to 0.27, *p* = 0.56), intervention duration (*β* = 0.05, 95% *CI*: −0.01 to 0.12, *p* = 0.11), training frequency (*β* = 0.07, 95% *CI*: −0.92 to 1.06, *p* = 0.89), average training duration per session (*β* = 0.11, 95% *CI*: −0.08 to 0.30, *p* = 0.25), and total weekly training duration (*β* = −0.00, 95% *CI*: −0.02 to 0.01, *p* = 0.65). The results demonstrated that none of the aforementioned continuous variables exerted a statistically significant moderating effect on the differential efficacy between CE and AE in reducing BFP (*p* > 0.05).

#### WC

**Figure 7:**
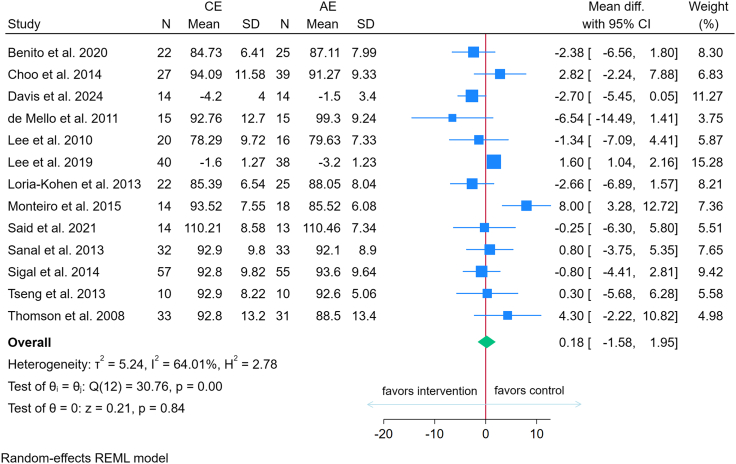
Meta-analysis results for waist circumference The forest plot shows the pooled effect of combined exercise compared with aerobic exercise on post-intervention waist circumference. The analysis included 13 independent studies and showed no statistically significant between-group difference. The pooled mean difference was 0.18 cm, with a 95% confidence interval from −1.58 to 1.95. Between-study heterogeneity was moderate to substantial, with τ^2^ = 5.24, I^2^ = 64.01%, and H^2^ = 2.78. Each square represents the effect estimate from an individual study, with square size proportional to study weight. Horizontal lines represent 95% confidence intervals, and the diamond represents the pooled random-effects estimate. Negative values indicate a greater reduction in waist circumference in the combined exercise group, whereas positive values indicate a greater reduction in the aerobic exercise group. Abbreviations: CE, combined exercise; AE, aerobic exercise; WC, waist circumference; MD, mean difference; CI, confidence interval; SD, standard deviation; REML, restricted maximum likelihood; τ^2^, between-study variance; I^2^, proportion of total variability due to between-study heterogeneity; H^2^, relative excess in Q statistic over its degrees of freedom; Q, Cochran’s heterogeneity statistic.

The meta-analysis, incorporating 13 independent studies, showed no statistically significant between-group difference between CE and AE in post-intervention waist circumference among adolescents and adults with overweight or obesity (MD = 0.18 cm, 95% CI: −1.58 to 1.95, *p* = 0.84). Between-study heterogeneity was moderate to substantial (τ^2^ = 5.24, I^2^ = 64.01%, H^2^ = 2.78; Q(12) = 30.76, *p* < 0.001) (Figure 7).

Exploratory subgroup analysis for WC (Table S6) suggested that CE was associated with a more favorable WC reduction than AE in moderate-intensity protocols. Regarding training sequence, fixed unidirectional CE protocols, either aerobic-prior-to-resistance or resistance-prior-to-aerobic, appeared less favorable than isolated AE for WC reduction. Furthermore, no significant subgroup effects were observed concerning age, BMI, intervention duration, training frequency, average training duration per session, or total weekly training duration (*p* > 0.05).

Meta-regression analyses were conducted to evaluate the potential moderating effects of the following continuous variables on the pooled main effect: age (*β* = −0.11, 95% *CI*: −0.26 to 0.04, *p* = 0.16), baseline BMI (*β* = −0.01, 95% *CI*: −0.50 to 0.48, *p* = 0.96), intervention duration (*β* = −0.02, 95% *CI*: −0.21 to 0.17, *p* = 0.82), training frequency (*β* = 0.80, 95% *CI*: −2.09 to 3.70, *p* = 0.59), average training duration per session (*β* = 0.14, 95% *CI*: −0.39 to 0.67, *p* = 0.60), and total weekly training duration (*β* = 0.01, 95% *CI*: −0.05 to 0.07, *p* = 0.81). The results demonstrated that none of the aforementioned continuous variables exerted a statistically significant moderating effect on the differential efficacy between CE and AE in reducing WC (*p* > 0.05).

#### LBM

**Figure 8:**
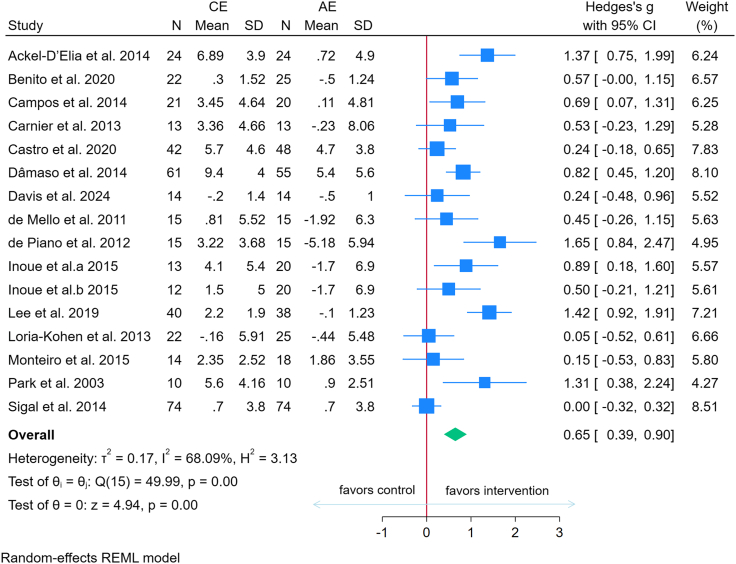
Meta-analysis results for lean body mass The forest plot shows the pooled effect of combined exercise compared with aerobic exercise on lean body mass. The analysis included 16 independent studies and indicated that combined exercise was associated with a greater increase in lean body mass than aerobic exercise. The pooled effect size was Hedges’ g = 0.65, with a 95% confidence interval from 0.39 to 0.90. Between-study heterogeneity was moderate to high, with I^2^ = 68.09%. Each square represents the effect estimate from an individual study, with square size proportional to study weight. Horizontal lines represent 95% confidence intervals, and the diamond represents the pooled random-effects estimate. Positive values indicate a greater increase in lean body mass in the combined exercise group, whereas negative values indicate a greater increase in the aerobic exercise group. Abbreviations: CE, combined exercise; AE, aerobic exercise; LBM, lean body mass; CI, confidence interval; SD, standard deviation; REML, restricted maximum likelihood; I^2^, proportion of total variability due to between-study heterogeneity; Hedges’ g, standardized mean difference corrected for small-sample bias.

The meta-analysis, incorporating 16 independent studies, suggested that CE was associated with a greater increase in LBM than AE among adolescents and adults with overweight or obesity (Hedges’ g = 0.65, 95% CI: 0.39 to 0.90, *p* < 0.001). Furthermore, a moderate-to-high degree of significant heterogeneity was observed across the included trials (*I*^*2*^ = 68.09%, *p* < 0.001) (Figure 8).

Exploratory subgroup analysis for LBM (Table S7) suggested that larger effects may be observed in studies with an average session duration ≥60 min, total weekly training duration ≥180 min, or alternating training sequences. However, these subgroup findings should be interpreted cautiously because several strata included a limited number of studies and subgroup analyses were not adjusted for multiplicity. No significant subgroup effects were observed concerning age, BMI classification, intervention duration, training frequency, or exercise intensity (*p* > 0.05).

Meta-regression analyses were conducted to evaluate the potential moderating effects of the following continuous variables on the pooled main effect: age (*β* = −0.01, 95% *CI*: −0.04 to 0.01, *p* = 0.37), baseline BMI (*β* = 0.01, 95% *CI*: −0.06 to 0.08, *p* = 0.82), intervention duration (*β* = 0.01, 95% *CI*: −0.01 to 0.03, *p* = 0.27), training frequency (*β* = 0.08, 95% *CI*: −0.33 to 0.48, *p* = 0.72), average training duration per session (*β* = 0.01, 95% *CI*: −0.06 to 0.09, *p* = 0.77), and total weekly training duration (*β* = 0.00, 95% *CI*: −0.00 to 0.01, *p* = 0.29). Meta-regression did not identify statistically significant associations between the examined continuous variables and the comparative LBM effect of CE versus AE (*p* > 0.05).

### Secondary cardiometabolic outcomes

For secondary cardiometabolic outcomes, including SBP, DBP, TC, TG, LDL-C, and HDL-C, we retained the overall pooled effects and a concise summary of exploratory subgroup and meta-regression analyses in the main text. The corresponding forest plots are provided in Figures S1, S2, S7, S8, S10, and S11; detailed subgroup analyses are provided in Tables S8, S9, S12, S13, S14, and S15; and the TG meta-regression results are provided in Figure S9.

For blood pressure outcomes, CE did not show a statistically significant advantage over AE for SBP. The pooled effect was MD = −2.19 mmHg, 95% CI: −4.59 to 0.21, *p* = 0.07, with moderate heterogeneity. Subgroup analyses did not identify significant subgroup differences according to age group, BMI classification, intervention duration, training frequency, training sequence, or exercise intensity. Meta-regression analyses also showed that age, baseline BMI, intervention duration, training frequency, and total weekly training duration were not significantly associated with the comparative effect of CE versus AE on SBP. Therefore, the current evidence does not support a clear additional effect of CE over AE for SBP reduction.

For DBP, CE showed a small statistically significant between-group difference compared with AE; however, the absolute magnitude was modest. The pooled effect was MD = −2.02 mmHg, 95% CI: −3.92 to −0.12, *p* = 0.04, with moderate heterogeneity. Exploratory subgroup analyses suggested that the DBP-lowering effect favoring CE was more evident in adult cohorts and in moderate-intensity exercise protocols. However, no significant subgroup effects were observed for BMI classification, intervention duration, training frequency, or training sequence. Meta-regression analyses showed that age was close to statistical significance as a potential moderator, whereas baseline BMI, intervention duration, training frequency, and total weekly training duration were not significant moderators. Given the small absolute effect size, limited study numbers, and low-certainty evidence, this secondary DBP finding should be interpreted as exploratory and of limited clinical relevance rather than as a clinically important advantage of CE.

For lipid outcomes, CE did not show consistent superiority over AE for TC, TG, or LDL-C. For TC, the pooled effect was MD = 0.04 mmol/L, 95% CI: −0.07 to 0.16, *p* = 0.46, with negligible heterogeneity. Subgroup analyses showed no significant subgroup differences according to age group, BMI classification, intervention duration, training frequency, average session duration, training sequence, or exercise intensity. Meta-regression analyses also showed no significant associations between TC effects and age, baseline BMI, intervention duration, training frequency, average session duration, or total weekly training duration. These findings indicate that CE and AE had broadly comparable effects on TC.

For TG, the pooled effect was not statistically significant, with MD = 0.02 mmol/L, 95% CI: −0.05 to 0.09, *p* = 0.62, and low heterogeneity. Subgroup analysis suggested that BMI classification may influence the direction of effect: CE appeared more favorable in overweight cohorts, whereas AE appeared more favorable in obese cohorts. Other subgroup variables, including age group, intervention duration, training frequency, average session duration, training sequence, and exercise intensity, did not show significant subgroup effects. Meta-regression analyses indicated that age and baseline BMI were significant moderators of the TG effect. Specifically, with increasing age, the comparative effect of CE on TG reduction became more favorable; in contrast, higher baseline BMI was associated with a less favorable comparative effect of CE. However, because these findings were based on study-level exploratory analyses and involved multiple comparisons, they should be interpreted as hypothesis-generating only.

For LDL-C, the pooled effect showed no statistically significant difference between CE and AE, with MD = 0.03 mmol/L, 95% CI: −0.07 to 0.13, *p* = 0.54, and negligible heterogeneity. Subgroup analyses did not show significant differences across age group, BMI classification, intervention duration, training frequency, average session duration, training sequence, or exercise intensity. Meta-regression analyses also showed no significant moderating effects of age, baseline BMI, intervention duration, training frequency, average session duration, or total weekly training duration. Thus, the available evidence suggests that CE and AE have similar effects on LDL-C.

For HDL-C, post-intervention HDL-C levels were modestly lower in the CE group than in the AE group, with MD = −0.03 mmol/L, 95% CI: −0.06 to −0.00, *p* = 0.03. Because the effect size was calculated as CE minus AE and higher HDL-C is generally considered favorable, this finding indicates a small advantage for AE for this lipid outcome. Subgroup analyses did not identify significant subgroup differences by age group, BMI classification, intervention duration, training frequency, average session duration, training sequence, or exercise intensity. Meta-regression analyses also did not show significant associations between HDL-C effects and the examined continuous moderators. Therefore, although AE showed a small advantage for HDL-C, the magnitude of this difference was small and should not be interpreted as evidence that AE is superior for the overall lipid profile.

Overall, the secondary outcome analyses found a small exploratory difference for DBP favoring CE and a small difference for HDL-C favoring AE; however, these findings should not be interpreted as clinically important advantages. No consistent between-group differences were observed for SBP, TC, TG, or LDL-C. Subgroup and meta-regression analyses suggested possible moderation by age and baseline BMI for TG, and possible stronger DBP effects in adults and moderate-intensity protocols. Nevertheless, because these analyses were exploratory, involved multiple comparisons, and were partly based on limited numbers of studies within subgroups, all moderator findings should be interpreted cautiously and considered hypothesis-generating rather than confirmatory.

### Sensitivity analysis and publication bias

**Figure 9:**
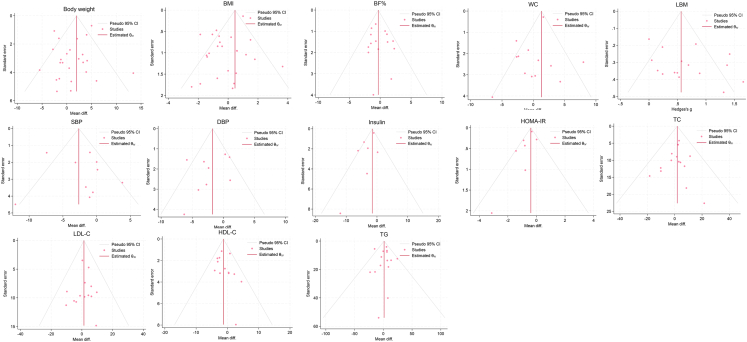
Funnel plot of publication bias The funnel plots were used to visually assess potential small-study effects and publication bias across the assessed body composition and cardiometabolic outcomes. Each point represents an individual study effect estimate. Visual inspection of the funnel plots, together with Egger’s linear regression tests, did not provide clear evidence of small-study effects for the assessed outcomes. However, these findings should be interpreted cautiously because the statistical power of funnel plot asymmetry tests may be limited when the number of studies is small for some outcomes. Abbreviations: BW, body weight; BMI, body mass index; BFP, body fat percentage; WC, waist circumference; LBM, lean body mass; SBP, systolic blood pressure; DBP, diastolic blood pressure; TC, total cholesterol; TG, triglycerides; LDL-C, low-density lipoprotein cholesterol; HDL-C, high-density lipoprotein cholesterol; HOMA-IR, homeostatic model assessment of insulin resistance.

A leave-one-out sensitivity analysis was conducted by sequentially omitting individual included studies. The sensitivity analyses did not materially change the magnitude or direction of the pooled estimates for body composition and cardiometabolic outcomes, suggesting that the main findings were relatively stable. Furthermore, both qualitative evaluation via visual inspection of funnel plots and quantitative assessment using Egger’s linear regression test did not provide clear evidence of small-study effects for the assessed outcomes, although the power of these analyses may have been limited for outcomes with few studies (*p* > 0.05, Figure 9).

### Summary of evidence

Based on the Grading of Recommendations Assessment, Development, and Evaluation (GRADE) system, the overall certainty of evidence for the core outcome indicators in this study ranged from very low to moderate (Table 1). Specifically, the certainty of evidence for the HDL-C outcome was rated as moderate quality; however, the observed direction favored AE rather than CE. The certainty of evidence concerning interventions for BFP, LBM, DBP, insulin, HOMA-IR, TC, TG, and LDL-C was evaluated as low quality. Furthermore, the evidence for BW, BMI, WC, and SBP was downgraded to very low quality.

**Table 1:** Evidence grade of the conclusion based on GRADE

Outcome measure	Risk of bias	Inconsistency	Indirectness	Imprecision	Publication bias	Quality of evidence
BW	−1	−1	0	−1	0	Very Low
BMI	−1	−1	0	−1	0	Very Low
BFP	−1	0	0	−1	0	Low
WC	−1	−1	0	−1	0	Very Low
LBM	−1	−1	0	0	0	Low
SBP	−1	−1	0	−1	0	Very Low
DBP	−1	−1	0	0	0	Low
Insulin	−1	−1	0	0	0	Low
HOMA-IR	−1	−1	0	0	0	Low
TC	−1	0	0	−1	0	Low
TG	−1	0	0	−1	0	Low
LDL-C	−1	0	0	−1	0	Low
HDL-C	−1	0	0	0	0	Moderate

This table summarizes the certainty of evidence for each outcome according to the GRADE framework. The certainty of evidence was assessed across five domains: risk of bias, inconsistency, indirectness, imprecision, and publication bias. A value of −1 indicates downgrading by one level for the corresponding domain, whereas 0 indicates no downgrading. The overall certainty of evidence ranged from very low to moderate across outcomes. The certainty of evidence was rated as very low for body weight, body mass index, waist circumference, and systolic blood pressure; low for body fat percentage, lean body mass, diastolic blood pressure, insulin, HOMA-IR, total cholesterol, triglycerides, and LDL-C; and moderate for HDL-C.

## Discussion

### Summary of primary findings

A pooled analysis of 29 RCTs showed that, compared with AE alone, CE was associated with greater improvements in LBM and selected insulin-resistance indicators, including insulin and HOMA-IR. However, CE did not demonstrate consistent superiority across several clinically important outcomes, including BW, BMI, BFP, WC, SBP, TC, TG, and LDL-C, and post-intervention HDL-C showed a small advantage for AE. Therefore, the present findings support CE as a potentially useful exercise option for selected body composition and metabolic outcomes, rather than as a universally superior or core intervention strategy for all cardiometabolic risk factors.

This study suggests that CE may be associated with greater improvements in LBM than AE, which is broadly consistent with findings from previous studies. The study by O'Donoghue et al.^30^ showed that combined high-intensity AT and high-load RT training is superior to any other exercise modality in improving LBM in obese adults. Similarly, García-Hermoso et al.^20^ found that in the obese pediatric population, compared with AT alone, CE can bring about greater LBM growth. In terms of glycemic control, our findings suggest that CE may be associated with greater reductions in insulin and HOMA-IR than AE; this pattern is consistent with previous evidence indicating that CE may improve insulin-resistance markers.^3^ Furthermore, Jamka et al.^31^ reported that CE was associated with reductions in HOMA-IR compared with endurance training.

Despite the commonalities in LBM and glycemic control, discrepancies exist regarding total adiposity and lipid metabolism indicators. This study found no significant differences between CE and AE in reducing BW, BFP, or LDL-C. In contrast, the research report by García-Hermoso et al.^20^ indicated that compared with AE alone, CE has better effects in reducing BW, fat mass, and LDL-C. Additionally, although we did not observe extra benefits of CE on TG, JamkaDE et al.^31^ found that combined training can significantly reduce TG levels compared to endurance training. Moreover, O'Donoghue et al.^30^ concluded that combined training has more advantages in reducing abdominal fat, whereas our analysis showed that the two modalities have comparable effects on WC.

Recent systematic reviews in populations with metabolic dysregulation further help contextualize our findings. In patients with T2DM and overweight/obesity, CE has been reported to improve glycemic control, blood pressure, inflammation, cardiorespiratory fitness, and quality of life, supporting the broader cardiometabolic relevance of combining AE and RT.^63^ Another recent meta-analysis also showed that CE improved BFP, lipid metabolism, and physical function in patients with T2DM and overweight/obesity, although no significant improvements were observed for body mass, waist-to-hip ratio, fat mass, or LBM when compared with standard treatment.^64^ Evidence for AE alone also supports its beneficial effects on BMI, WC, BFP, glucose metabolism, HDL-C, TG, and TC in patients with T2DM and overweight/obesity, indicating that AE remains an important non-pharmacological strategy for cardiometabolic risk management.^65^ In addition, HIIT has been shown to improve several metabolic and cardiovascular risk markers in patients with T2DM and obesity, including body mass, BMI, fasting glucose, HbA1c, fasting insulin, HOMA-IR, LDL-C, TG, and TC, even when changes in body composition are limited.^66^ When HIIT was compared directly with moderate-intensity continuous training, HIIT showed modest advantages for fasting insulin, HOMA-IR, and cardiorespiratory fitness, whereas body composition, lipid profile, and blood pressure showed broadly comparable responses between modalities.^67^ Taken together, these recent findings indicate that CE, AE, RT-containing programs, and HIIT may each contribute to cardiometabolic health in metabolically dysregulated populations, but their benefits appear to differ across outcome domains. This aligns with our results suggesting that CE may be more favorable for LBM and selected metabolic indicators, whereas consistent superiority over AE was not observed across adiposity, blood pressure, and lipid outcomes.

### Sources of heterogeneity and interpretation of pooled estimates

The pooled estimates should be interpreted in light of the clinical and methodological heterogeneity across the included trials. Participants differed substantially in age, developmental stage, baseline BMI, and cardiometabolic disease status. These factors may influence exercise responsiveness because adolescents and adults differ in growth-related adaptations, hormonal status, muscle hypertrophic potential, insulin sensitivity, and vascular function. In addition, some trials included participants with uncomplicated overweight or obesity, whereas others enrolled individuals with hypertension, T2DM, or metabolic syndrome, which may have affected baseline risk profiles and the magnitude of cardiometabolic responses.

Variability in intervention design may also have contributed to between-study differences. CE and AE protocols differed in intervention duration, weekly frequency, session length, total training volume, exercise intensity, training sequence, progression strategy, supervision, and adherence monitoring. Therefore, the pooled effects should not be interpreted as the effect of a single standardized CE prescription, but as an average estimate across diverse populations and exercise protocols. These sources of heterogeneity may partly explain why CE showed clearer benefits for LBM and insulin-resistance indicators, whereas the small DBP difference should be viewed as exploratory and of limited clinical relevance. Accordingly, subgroup and meta-regression findings should be considered exploratory and hypothesis-generating.

### Differential effects of body composition indicators and analysis of moderating factors

Among body composition indicators, CE did not demonstrate superior effects in reducing BW, BMI, or WC compared to AE, which is partially consistent with previous network meta-analyses.^21^ However, subgroup analyses revealed several key moderating factors. In short-term interventions with a duration of ≤12 weeks, CE showed significantly greater weight loss effects than AE; however, as the intervention period extended, AE exhibited better outcomes. This may be attributable to decreased adherence to combined protocols or adaptive responses in participants during long-term exercise interventions.^68^ Although meta-regression analysis of BW did not identify an overall moderating effect of age, regression analysis of BMI indicated a significant moderating role of age: the advantage of CE in reducing BMI became more pronounced with increasing age, particularly in adult populations.^69^^,^^70^ This suggests that the difference in BMI effects between the two exercise modalities is smaller in young individuals, whereas adults may derive greater benefits from CE. Furthermore, exercise intensity significantly influenced the outcomes for BMI, BFP, and WC: under moderate-intensity interventions, CE was superior to AE; however, under high-intensity conditions, this advantage disappeared or even reversed.^71^ This implies that when training intensity is too high, interference effects between AE and RT may occur, thereby attenuating the additive benefits of CE.

Furthermore, exercise intensity appeared to influence the outcomes for BMI, BFP, and WC: under moderate-intensity interventions, CE showed more favorable effects than AE; however, under vigorous/high-intensity conditions, this advantage disappeared or even reversed.^71^ This pattern may be partly related to interference effects between AE and RT when training intensity is high, thereby attenuating the additive benefits of CE. However, the interpretation of intensity-related subgroup findings should be cautious because exercise intensity was operationalized differently across trials, including heart rate-based, oxygen uptake-based, perceived exertion-based, ventilatory threshold-based, and resistance load-based indicators. Therefore, the observed differences between moderate- and vigorous/high-intensity protocols should be regarded as exploratory rather than definitive evidence for an optimal training intensity.

### The enhancing effect of CE on LBM

CE was associated with a greater increase or preservation of LBM than AE, a finding that may be clinically relevant for maintaining metabolic health.^72^ Exploratory subgroup analyses suggested that longer session duration, greater weekly training volume, and alternating training sequences may be linked to larger LBM effects; however, these findings should not be interpreted as definitive dose or sequencing recommendations.^73^^,^^74^

### Outcome-specific effects on cardiometabolic risk indicators

Regarding cardiometabolic risk indicators, CE showed statistically significant differences favoring insulin and HOMA-IR. A small difference in DBP also favored CE, but this was a secondary outcome with limited clinical relevance. However, HDL-C showed the opposite direction, with slightly higher post-intervention HDL-C levels in the AE group than in the CE group. This finding should be interpreted cautiously because the absolute between-group difference was small and was based on post-intervention values rather than necessarily indicating greater within-group improvement in either exercise modality. In addition, no significant between-group differences were observed for TC, TG, or LDL-C, suggesting that the comparative effects of CE and AE on lipid outcomes remain inconsistent. The DBP finding appeared more evident in adult populations and moderate-intensity protocols, but these subgroup patterns should be interpreted cautiously.^75^ For insulin-resistance-related indicators, the pooled results suggested potentially favorable effects of CE compared with AE.^76^ Subgroup and meta-regression analyses indicated possible variation by age and intervention duration, but these patterns should be regarded as exploratory because of limited study numbers and between-study heterogeneity.^20^^,^^77^ These findings suggest that CE may be a promising option for improving insulin resistance-related markers in younger populations with overweight or obesity.^78^ Nevertheless, because the included trials did not assess long-term diabetes incidence or disease progression, CE should not be interpreted as a proven strategy for preventing progression from insulin resistance to T2DM. However, no differences were observed between the two exercise modalities in terms of TC, TG, or LDL-C,^79^ indicating that improvements in lipid metabolism may depend more on exercise-induced total BW and fat loss rather than on the specific training modality.^80^

### Potential physiological explanations and their interpretive limits

The observed differences between CE and AE in LBM and insulin-resistance markers may be biologically plausible. The small DBP finding is discussed only as an exploratory secondary observation, and the underlying mechanisms were not directly tested in the included trials. Therefore, the following explanations should be considered hypothesis-generating. The RT component effectively activates the mammalian target of rapamycin signaling pathway, directly stimulating skeletal muscle protein synthesis and inducing muscle hypertrophy.^81^ Increased LBM is not only a major determinant of resting energy expenditure but also a key site for glucose storage and oxidation.^82^ Exploratory subgroup analyses suggested that larger LBM effects may occur when sessions last ≥60 min and when alternating training sequences are used, but these findings do not confirm an optimal prescription or mechanism.^83^ Second, the improvement in insulin resistance represents another core metabolic benefit of CE. Although AE can improve glucose metabolism by increasing the expression of insulin sensitivity-related proteins,^84^ CE may confer additive advantages through a mass effect: the increased skeletal muscle mass itself provides a larger reservoir for glucose uptake,^85^ thereby enhancing peripheral insulin sensitivity independently of total BW or fat loss.^86^ Meta-regression suggested that the comparative effect of CE on HOMA-IR may become more favorable with longer intervention duration, although this pattern should be interpreted cautiously.^87^ Because no consistent differences were observed for BMI or BFP, the insulin-resistance findings may reflect mechanisms beyond overall fat reduction, but this interpretation remains indirect and hypothesis-generating.^88^^,^^89^

In terms of cardiovascular regulation, the improvement in DBP with CE may be partly explained by the complementary vascular adaptations induced by aerobic and resistance exercise. Previous meta-analytic evidence indicates that endurance, dynamic resistance, and combined training can reduce resting blood pressure, with combined training showing a significant effect on DBP.^75^ Exercise training may also improve endothelial function, as aerobic, resistance, and CE modalities have all been shown to enhance flow-mediated dilation, a commonly used marker of endothelial function.^90^ Therefore, the DBP-lowering effect observed in the present study may plausibly reflect improvements in vascular tone, peripheral resistance, and endothelial function; however, these mechanisms were not directly measured in the included trials and should be interpreted as biologically plausible rather than confirmed. Because AE showed a small advantage over CE for post-intervention HDL-C, no mechanistic inference supporting a specific HDL-C benefit of CE can be drawn from the present meta-analysis. The inconsistent findings across HDL-C, TC, TG, and LDL-C suggest that lipid-related responses may depend on factors beyond exercise modality alone, including baseline metabolic status, total energy expenditure, dietary control, and intervention adherence.^91^^,^^92^ Finally, the influence of training sequence on outcomes provides a plausible explanation for some subgroup differences. Evidence from concurrent training research suggests that exercise order can influence acute resistance performance and longer-term adaptations, and that interference effects may depend on how endurance and resistance stimuli are sequenced.^83^^,^^93^ Therefore, the observed advantages of alternating training sequences for LBM and WC should be regarded as hypothesis-generating rather than definitive mechanistic evidence.

Overall, the observed advantages of CE over AE alone for LBM, insulin resistance, and DBP may be supported by several physiologically plausible pathways, including anabolic adaptations in skeletal muscle, greater skeletal-muscle glucose disposal, and vascular adaptations related to endothelial function and peripheral resistance. However, these mechanisms were not directly measured in the included trials. Therefore, explanations involving mTOR activation, endothelial function, vascular tone, and lipid metabolism should be interpreted as biologically plausible and hypothesis-generating rather than as confirmed mechanistic evidence within the scope of this meta-analysis.

This meta-analysis suggests that, compared with AE alone, CE may provide outcome-specific benefits for increasing LBM and improving selected insulin-resistance indicators. However, CE did not show consistent superiority across adiposity-related outcomes, blood pressure outcomes, or lipid profiles. However, CE did not show consistent superiority across adiposity-related outcomes, SBP, or lipid profiles, and AE showed a small advantage for post-intervention HDL-C. Given the low to very low certainty of evidence for most outcomes, these findings should be interpreted cautiously, and CE should not be regarded as a universally superior or core strategy. Future high-quality RCTs with standardized exercise prescriptions, longer follow-up, and direct assessment of clinical and mechanistic outcomes are needed to clarify when and for whom CE offers meaningful advantages over AE.

### Limitations of the study

The limitations of this study include a high risk of bias concerning the blinding of participants and personnel, which is inherent to the nature of exercise interventions. Significant methodological heterogeneity exists across individual studies in terms of prescription elements such as session duration, intensity range, and sequencing, as well as baseline characteristics including age range and obesity degree, which partially limits the generalizability of the pooled effects. Additionally, while we categorized primary and secondary outcomes to structure our analysis, the extensive use of subgroup and meta-regression analyses introduces the risk of Type I errors due to multiplicity. Although these analyses provide valuable insights into moderating factors, they remain inherently exploratory. Furthermore, the existing literature largely focuses on short-to medium-term physiological adaptations, lacking longitudinal tracking of long-term adherence and hard cardiovascular endpoints.

Future high-quality RCTs should improve design standardization, strictly adhere to the FITT principle, and accurately report total work and metabolic load to elucidate optimal dose-response relationships and explore optimal sequencing strategies that avoid the interference effect of concurrent training. Subsequent research should rigorously control dietary confounding factors, unify outcome measurement indicators, and place emphasis on long-term follow-up and hard cardiovascular endpoints, thereby effectively reducing clinical heterogeneity and providing high-level evidence-based support for the development of individualized exercise prescriptions.

In addition, the clinical implications of CE should be interpreted cautiously. Although CE improved LBM and selected insulin resistance-related outcomes, the absence of consistent superiority across adiposity, SBP, and lipid outcomes, together with the low to very low certainty of evidence for most endpoints, limits any recommendation of CE as a universal core strategy.

## Resource availability

### Lead contact

Further information and requests should be directed to and will be fulfilled by the Lead Contact, Ming Li, baomei005@163.com.

### Materials availability

This study did not generate new unique reagents or materials.

### Data and code availability

All data extracted and analyzed in this study are available in the article and supplemental information. Additional information is available from the Lead Contact upon reasonable request.

## Acknowledgments

This study was funded by the 10.13039/501100012456National Social Science Foundation Late-stage Funding General Project “Research on the Development Model and Path Optimization of Equitable and Accessible Public Sports Services in China” project no. 24FTYB002.

## Author contributions

J.Y. and Y.H. conceived and designed the study. J.Y. and Y.H. conducted the literature search and study selection. J.Y. and H.L. extracted the data. H.K. and H.L. assessed the certainty of evidence using the GRADE approach. J.Y. performed the statistical analyses and drafted the manuscript. Y.H., H.L., H.K., and M.L. critically revised the manuscript for important intellectual content. M.L. supervised the study. All authors read and approved the final manuscript.

## Declaration of interests

The authors declare no competing interests.

## STAR★Methods

### Key resources table

**Table:** 

REAGENT or RESOURCE	SOURCE	IDENTIFIER
**Deposited data**		

PROSPERO registration	PROSPERO	PROSPERO: CRD420261338172

**Software and algorithms**		

Stata 17.0	StataCorp LLC	https://www.stata.com
Review Manager 5.4	Cochrane	https://training.cochrane.org/online-learning/core-software/revman
GetData Graph Digitizer	GetData Pty Ltd	http://getdata-graph-digitizer.com
EndNote	Clarivate	https://endnote.com
Cochrane risk-of-bias tool for randomized trials RoB 2	Cochrane Methods	https://www.riskofbias.info/welcome/rob-2-0-tool
GRADE approach	GRADE Working Group	https://www.gradeworkinggroup.org

**Other**		

PubMed	National Library of Medicine	https://pubmed.ncbi.nlm.nih.gov
Scopus	Elsevier	https://www.scopus.com
Embase	Elsevier	https://www.embase.com
Web of Science	Clarivate	https://www.webofscience.com
EBSCO	EBSCO Information Services	https://www.ebsco.com
ProQuest	Clarivate	https://www.proquest.com
Cochrane Library	Cochrane	https://www.cochranelibrary.com
PRISMA 2020 guidelines	PRISMA Statement	https://www.prisma-statement.org
Cochrane Handbook for Systematic Reviews of Interventions	Cochrane	https://training.cochrane.org/handbook
American College of Sports Medicine exercise intensity criteria	American College of Sports Medicine	ACSM Guidelines for Exercise Testing and Prescription
World Health Organization physical activity guidelines	World Health Organization	WHO guidelines on physical activity and sedentary behavior

### Method details

#### Literature search strategy

The protocol for this systematic review and meta-analysis was prospectively registered with the PROSPERO database (Registration number: CRD420261338172), and both the literature search and execution of this study were conducted in strict accordance with the Preferred Reporting Items for Systematic Reviews and Meta-Analyses (PRISMA) guidelines.^94^ Two investigators (JY and YH) independently performed a comprehensive literature search across the following databases: PubMed, Scopus, Embase, Web of Science, EBSCO, ProQuest, and the Cochrane Library. The systematic retrieval encompassed all randomized controlled trials published from database inception up to January 2026 that investigated the comparative effects of CE versus isolated AE.

The search strategy was meticulously tailored to the specific syntactic requirements of each database, integrating controlled vocabulary (e.g., MeSH terms) and free-text keywords. The core search logic was constructed around three primary domains: the target population (“Obesity”, “Overweight”, “Adolescent”, “Young Adult”), the interventions (“Combined exercise”, “Concurrent training”, “Aerobic exercise”, “Resistance training”), and the outcome measures (“Body composition”, “Cardiometabolic risk”, “Insulin resistance”, “Lipid profile”). To maximize search comprehensiveness and minimize omission rates, no language or publication status restrictions were imposed. Additionally, a manual snowballing technique was employed to scrutinize the reference lists of previously published systematic reviews and the finally included studies. All retrieved bibliographic records were subsequently imported into the EndNote reference management software for deduplication, followed by blinded screening.

#### Study selection

The study selection process was executed in rigorous adherence to the PRISMA statement. Following the removal of duplicate records utilizing EndNote reference management software, two investigators (JY and YH) independently screened the titles and abstracts of the retrieved articles to identify potentially eligible studies. Subsequently, full texts of all potentially relevant articles were retrieved and subjected to an independent, in-depth evaluation against the predefined inclusion and exclusion criteria to ascertain final eligibility. Any discrepancies or disagreements arising during the screening phases were resolved through rigorous cross-checking and consensus between the two primary reviewers; unresolved conflicts were adjudicated by a third senior investigator (HK).

#### Inclusion and exclusion criteria

Inclusion and exclusion criteria were formulated in strict accordance with the PICOS framework. The inclusion criteria were stipulated as follows: (1) Participants were eligible if they were aged 6–45 years and had overweight or obesity defined using BMI-based diagnostic criteria. According to PubMed/MeSH age categories, this age range includes children aged 6–12 years, adolescents aged 13–18 years, and adults aged 19–44 years. Participants aged 45 years were retained when the original trial defined the cohort as adults and the mean age remained within the adult range. For participants younger than 18 years, overweight and obesity were defined using age- and sex-specific BMI criteria reported in the original trials, including WHO BMI-for-age references or IOTF cut-offs. For adults, overweight and obesity were defined as BMI ≥25 kg/m^2^ and BMI ≥30 kg/m^2^, respectively. Studies including participants with obesity-related comorbidities, such as hypertension, type 2 diabetes mellitus, metabolic syndrome, or polycystic ovary syndrome, were eligible when the primary condition was overweight or obesity and when CE and AE were compared under comparable intervention conditions; (2) the intervention protocol must employ a clearly defined CE for the experimental group, whereas the control group must undergo isolated AE; (3) included studies must report at least one body composition or cardiometabolic risk parameter. In this study, outcome measures were categorized into primary and secondary indicators. Primary outcomes included BW, BMI, BFP, waist circumference (WC), LBM, insulin, and homeostasis model assessment of insulin resistance (HOMA-IR). Secondary outcomes comprised cardiovascular and lipid profile parameters, specifically: systolic blood pressure (SBP), diastolic blood pressure (DBP), total cholesterol (TC), triglycerides (TG), low-density lipoprotein cholesterol (LDL-C), and high-density lipoprotein cholesterol (HDL-C). Finally, (4) the study design was strictly restricted to RCT.

Conversely, the corresponding exclusion criteria encompassed: (1) non-RCT study designs, such as observational studies, cross-sectional surveys, case reports, animal experiments, conference abstracts, and reviews; (2) trials incorporating confounding interventions that could not be independently isolated, particularly where the control group failed to maintain equivalent baseline conditions; (3) studies with incomplete data reporting, severe logical discrepancies, or outcome data presented exclusively in graphical formats, where the raw mean and standard deviation required for effect size calculation remained unobtainable even after attempting to contact the original authors; and (4) duplicate publications derived from the same cohort that did not provide novel follow-up data or distinct outcome measures.

#### Data extraction and transformation

The data extraction and transformation procedures were independently executed by two specifically trained investigators (YH and HK) utilizing a pre-designed, standardized electronic data extraction form. The core extracted information comprehensively encompassed fundamental study characteristics, participant baseline demographics, intricate parameters of the intervention and control protocols, as well as the pre- and post-intervention means and standard deviations for all respective body composition and cardiometabolic risk outcome measures. In instances of missing data or results presented exclusively in graphical formats, the corresponding authors were contacted to acquire the requisite raw data. In the event of unsuccessful communication, data were digitally extracted from the available figures utilizing the GetData Graph Digitizer software.

When studies reported the standard error (SE), it was mathematically converted to the standard deviation (SD)^95^ using the formula:

$$SD=\sqrt{N}\times SE$$

For the comparative analysis of the changes from baseline between the intervention and control groups, the mean change was calculated using the following equation:

$$M_{change}=M_{post}-M_{pre}$$

where M_post_ and M_pre_ represent the post-intervention and pre-intervention means, respectively.

The SD of the mean change for both groups was imputed via the following equation:

$$SD_{change}=\sqrt{SD_{pre}^{2}+SD_{post}^{2}-\left(2\times R\times SD_{pre}\times SD_{post}\right)}$$

Here, SD_pre_ and SD_post_ denote the standard deviations before and after the intervention, respectively, and R represents the correlation coefficient between the baseline and post-intervention measurements. In accordance with the recommendations outlined in the Cochrane Handbook,^95^ an R value of 0.5 was conservatively imputed.

For studies involving multiple eligible intervention subgroups, the data were pooled into a single pairwise comparison. Assuming Subgroup A comprises a sample size of N_1_, mean of M_1_, and standard deviation of SD_1_, and Subgroup B comprises N_2_, M_2_, and SD_2_, the pooled sample size and pooled mean were calculated as N = N_1_ + N_2_ and M = (N_1_M_1_ + N_2_M_2_)/(N_1_ + N_2_), respectively. The pooled standard deviation (SD_pooled) was computed utilizing the following formula:

$$SD_{pooled}=\sqrt{\frac{\left(N_{1}-1\right)SD_{1}^{2}+\left(N_{2}-1\right)SD_{2}^{2}+\frac{N_{1}N_{2}}{N_{1}+N_{2}}\left(M_{1}^{2}+M_{2}^{2}-2M_{1}M_{2}\right)}{N_{1}+N_{2}-1}}$$

#### Risk of bias assessment

Two investigators (JY and YH) independently evaluated the methodological quality of the included RCTs utilizing the revised Cochrane risk-of-bias tool for randomized trials (RoB 2.0).^96^ The appraisal rigorously encompassed five fundamental domains: bias arising from the randomization process, bias due to deviations from intended interventions, bias due to missing outcome data, bias in measurement of the outcome, and bias in selection of the reported result. Within each respective domain, as well as for the overall risk of bias, studies were definitively categorized as having a “low risk of bias,” “some concerns,” or a “high risk of bias”. Any discrepancies emerging during the evaluation phase were resolved through rigorous cross-checking and consensus between the two primary reviewers; unresolved conflicts were subsequently adjudicated by a third senior investigator (HL).

#### Assessment of evidence quality

Two investigators (HL and HK) independently evaluated the overall quality of evidence for each primary outcome measure utilizing the Grading of Recommendations Assessment, Development and Evaluation (GRADE) system.^97^ Premised on five fundamental domains for downgrading—risk of bias, inconsistency, indirectness, imprecision, and publication bias—the certainty of evidence for each respective outcome was rigorously classified into one of four tiers: high, moderate, low, or very low. Any discrepancies emerging during the appraisal process were resolved through rigorous cross-checking and consensus; when deemed necessary, unresolved conflicts were independently adjudicated by a third senior investigator (JY).

### Quantification and statistical analysis

All statistical analyses were executed utilizing Stata 17.0 and Review Manager (RevMan) 5.4 software. For continuous outcome variables, the mean difference or standardized mean difference was adopted as the effect measure, with all pooled estimates presented alongside their 95% confidence intervals.^98^ Anticipating potential clinical and methodological heterogeneity across the included studies regarding exercise intervention protocols and participant characteristics, a random-effects model was *a priori* employed for all meta-analyses to yield more conservative estimates. Between-study heterogeneity was quantitatively appraised utilizing Cochran’s Q test in conjunction with the *I*^*2*^ statistic^99^: an *I*^*2*^ ≤ 50% coupled with a *p* ≥ 0.10 denoted acceptable heterogeneity; conversely, an *I*^*2*^ > 50% or a *p* < 0.10 was indicative of substantial heterogeneity. Under such conditions, pooled results were interpreted with caution, and subsequent subgroup analyses and meta-regression were planned to profoundly investigate the potential sources of heterogeneity.^100^ To explore potential moderating variables, subgroup analyses were stratified according to *a priori* established study characteristics, encompassing intervention duration, participant age group, degree of obesity, weekly training frequency, average training duration per session, total weekly training duration, training sequence, and exercise intensity.

Exercise intensity information was extracted from the original intervention descriptions and is summarized in Table S2. The extracted indicators included percentage of maximum heart rate (%HRmax), heart rate reserve (%HRR), oxygen uptake reserve or peak oxygen uptake (%VO_2_R/%VO_2_peak), ventilatory threshold, rating of perceived exertion (RPE), percentage of one-repetition maximum (%1RM), repetition maximum (RM) range, repetition targets, and author-defined intensity descriptions. Because the included trials reported exercise intensity using heterogeneous metrics, we harmonized intensity categories according to the American College of Sports Medicine (ACSM) exercise prescription criteria^101^^,^^102^ and the World Health Organization (WHO) physical activity guidelines.^103^

For aerobic exercise, moderate intensity was defined as 40%–59% HRR or VO_2_R/VO_2_peak, 64%–76% HRmax, ventilatory threshold-based moderate training, or RPE 12–13. Vigorous or high intensity was defined as ≥60% HRR or VO_2_R/VO_2_peak, ≥77% HRmax, or RPE ≥14. For resistance training, moderate intensity was defined as 50%–69% 1RM or approximately 12–20 RM, whereas vigorous or high intensity was defined as ≥70% 1RM or ≤10 RM.

When CE protocols included both aerobic and resistance components, classification was based on the dominant prescribed intensity or the highest target intensity reached during the main training phase. Progressive protocols spanning more than one category were classified according to the target intensity achieved during the main intervention period. Given the variability in intensity metrics, progression schemes, supervision, and adherence monitoring across studies, intensity subgroup analyses were considered exploratory and hypothesis-generating rather than confirmatory.

Provided an adequate number of included studies were available, random-effects meta-regression models were used to evaluate whether continuous variables, including age, baseline BMI, intervention duration, training frequency, average session duration, and total weekly training duration, moderated the pooled effects.^104^ To assess the robustness of the pooled estimates, leave-one-out sensitivity analyses were conducted by sequentially omitting each included study. Where feasible, we also examined whether the direction and magnitude of pooled effects were consistent across age and obesity subgroups. For outcomes including ≥10 studies, potential publication bias was evaluated by visual inspection of funnel plots^105^ and Egger’s linear regression test.^106^

Because multiple subgroup and meta-regression analyses were conducted across several outcomes, these analyses were considered exploratory and hypothesis-generating rather than confirmatory. Formal multiplicity correction was applied to the primary and secondary pooled outcomes using the False Discovery Rate correction (Benjamini-Hochberg procedure), but not to subgroup or meta-regression analyses because of limited study numbers within several strata. Therefore, nominal *p* values from subgroup and meta-regression analyses should be interpreted cautiously and were used primarily to identify potential sources of heterogeneity.

## Supplemental information

Document S1. Figures S1–S11 and Tables S1–S15

Data S1. Extracted data for meta-analysis, related to Methods and Results

Data S2. Search strategies for bibliographic databases, related to Methods
