# Supplementary material for: Combined exercise for body composition and cardiometabolic health in obese young people meta analysis
Source: iScience. 2026 Jul 21;29(8):116888. doi: 10.1016/j.isci.2026.116888 (PMC13392960; doi:10.1016/j.isci.2026.116888)
Supplement: Document S1. Figures S1–S11 and Tables S1–S15 [file mmc1.pdf]

iScience, Volume *29*

Supplemental information

Combined exercise for body composition and cardiometabolic health in obese young people meta analysis

Jingyou Zhong, Yanhao Wang, Hongliang Wu, Huangkun Chen, and Ming Li

Supplemental Table 1: Basic Characteristics of the Included Studies

Study	Year	Group	Sample Size (N)	(M/F)	Age	Baseline BMI (kg/m²)	Subject Condition	Intervention Measures	Body Composition Method	Reported Outcome Indicators
Ambelu et al.	2023	CE	10	7/3	42.60 ± 7.20	29.26 ± 2.94	T2DM	3 times/week, intensity RPE 11-13 and 40-70% HRR, 60 mins/session, including 30 mins resistance and 30 mins aerobic training	Skinfold thickness method	SBP, DBP, BMI, BFP
		AE	10	7/3	41.10 ± 7.22	28.45 ± 3.67		3 times/week, intensity 40-70% HRR, 60 mins/session, aerobic dance training		
Ackel-D'Elia et al.	2014	CE	24	9/15	16.46 ± 1.51	35.10 ± 4.67	Obesity	3 times/week, 60 mins/session, including 30 mins aerobic (heart rate corresponding to ventilatory threshold 1) and 30 mins resistance (10 exercises, 3 sets, 6-20 RM)	BOD POD	BW, BMI, LBM, INS, HOMA-IR
		AE	24	7/17	16.46 ± 1.51	35.06 ± 3.90		3 times/week, 60 mins/session, aerobic exercise on treadmill or cycle ergometer, intensity at heart rate corresponding to ventilatory threshold 1		
Alemayehu et al.	2023	CE	12	12/0	44.00 ± 7.97	28.93 ± 1.94	Hypertension	Intensity RPE 11-13 and 64-76% HRmax, 60 mins/session, including 30 mins resistance and 30 mins aerobic training	Skinfold thickness method	SBP, DBP, BW, BMI, BFP
		AE	11	11/0	45.64 ± 6.58	27.42 ± 1.85		Intensity 64-76% HRmax, 60 mins/session, aerobic dance training		
Benito et	2020	CE	22		37.4 ± 8.1	27.74 ± 1.12	Overweight	3 times/week, intensity 50-60% 15RM and 50-60% HRR, avg. 55	DXA	BW, BMI, WC, LBM

al.								mins/session, interleaved aerobic (run/bike/elliptical) and resistance (squat/rowing, etc.) training		
		AE	25		37.4 ± 8.1	28.64 ± 1.46		3 times/week, intensity 50-60% HRR, avg. 55 mins/session, running, cycling, or elliptical training		
Campos et al.	2014	CE	21	7/14	16.3 ± 1.53	37.6 ± 5.44	Obesity	3 times/week, 60 mins/session, including 30 mins aerobic (intensity at ventilatory threshold 1 HR ±4bpm) and 30 mins resistance (10 exercises, 3 sets, 6-20 RM with periodized alternation)	BOD POD	BW, BMI, LBM, INS, HOMA-IR
		AE	21	7/14	16.14 ± 1.17	35.82 ± 4.52		3 times/week, 60 mins/session, aerobic exercise on treadmill or cycle ergometer, intensity at ventilatory threshold 1 HR ±4bpm		
Carnier et al.	2013	CE	13	5/8	NR	34.41 ± 3.53	Obesity	3 times/week, 60 mins/session, first 30 mins aerobic (ventilatory threshold 1 HR ±4bpm), then 30 mins resistance (8 exercises, 3 sets, 6-20 RM periodized adjustment)	BOD POD	BW, BMI, LBM
		AE	13	5/8	NR	35.36 ± 3.95		3 times/week, 60 mins/session, aerobic exercise on treadmill, intensity at ventilatory threshold 1 HR ±4bpm		
Castro et al.	2020	CE	42	21/21	38.7 ± 6.6	30.2 ± 3.1	Obesity	3 times/week, 60 mins/session, first 30 mins aerobic (intensity increasing from VT1 to near VT2), then 30 mins resistance (8 exercises, intensity increasing from 15-20RM to 10-12RM), for 12 weeks	DXA	BW, BMI, BFP, LBM

		AE	48	20/28	37.4 ± 7.9	30.4 ± 2.6		3 times/week, circuit training, intensity 50-60% HRR, duration increasing from 51 min 15 sec to 64 min		
Chen et al.	2015	CE	15	15/0	14.2 ± 3.8	28.4 ± 2.8	Obesity	3 times/week, intensity 60% VO2max and 8-12RM, 60 mins/session, resistance followed by aerobic exercise	BIA	BW, BMI, BFP
		AE	15	15/0	14.1 ± 3.1	27.8 ± 3.0		3 times/week, intensity 60% VO2max, 60 mins/session, running training		
Choo et al.	2014	CE	27	0/27	41.8 ± 6.6	29.1 ± 0.79	Overweight	3 times/week, intensity 50-70% HRR and 8-12 reps, 60 mins/session, resistance followed by aerobic training	BIA	BW, WC, SBP, TC, LDL-C, HDL-C, TG
		AE	39	0/39	42.2 ± 9.5	28.5 ± 0.54		3 times/week, intensity 50-70% HRR, 60 mins/session, treadmill and bike training		
Dâmaso et al.	2014	CE	61		16.9 ± 1.8	36.7 ± 4.9	Obesity	3 times/week, intensity at ventilatory threshold I and to exhaustion (15-20RM changing to 6-20RM), 60 mins/session, alternating 30 mins aerobic and 30 mins resistance training	ADP	BW, BMI, BFP, LBM, INS, HOMA-IR, TC, HDL-C, LDL-C, TG
		AE	55		16.9 ± 1.8	35.7 ± 4.3		3 times/week, intensity at ventilatory threshold I (approx. 50-70% VO2peak), 60 mins/session, treadmill running training		
Davis et al.	2024	CE	17	0/17	37.7 ± 6.7	38.5 ± 6.7	Obesity	3 times/week, intensity 40-80% HRR and 40-80% 1RM, 50 mins/session, combined training of 25 mins aerobic and 3 resistance exercises	BIA	BMI, BW, BFP, WC
		AE	16	0/16	38.6 ± 8.6	39.7 ± 6.0		3 times/week, intensity 40-80% HRR, 50 mins/session, stationary bike or		

							elliptical training		
de Mello et al.	2011	CE	15	16.71 ± 1.47	37.23 ± 5.19	Metabolic Syndrome	3 times/week, intensity at ventilatory threshold I and 6-20RM (repetition maximum), 60 mins/session, alternating 30 mins aerobic and 30 mins resistance training	ADP	BW, BMI, LBM, TC, LDL-C, HDL-C, INS, HOMA-IR, TG, WC, SBP, DBP
		AE	15	16.71 ± 1.47	36.63 ± 4.14		3 times/week, intensity at ventilatory threshold I (approx. 50-70% VO2peak), 60 mins/session, treadmill running training		
de Piano et al.	2012	CE	15	16.48 ± 1.42	36.46 ± 4.49	Obesity	3 times/week, intensity at ventilatory threshold I (50-70% VO2peak) and 6-20 reps, 60 mins/session, alternating 30 mins aerobic and 30 mins resistance training	ADP	BW, BMI, INS, HOMA-IR, TC, LDL-C, HDL-C, TG, LBM
		AE	15	16.48 ± 1.42	33.53 ± 3.58		3 times/week, intensity at ventilatory threshold I (approx. 50-70% VO2peak), 60 mins/session, running or cycling training		
Inoue et al.	2015	CE	13	16.28 ± 1.34	36.4 ± 1.6	Obesity	3 times/week, intensity at ventilatory threshold I (VTI) and periodized RM (15-20/10-12/6-8RM), 60 mins/session, alternating 30 mins aerobic and 30 mins resistance training	ADP	BW, BMI, BFP, LBM, TG, TC, LDL-C
		CE	12	16.28 ± 1.34	38.2 ± 1.3		3 times/week, intensity at ventilatory threshold I (VTI) and daily undulating RM (15-20/10-12/6-8RM), 60 mins/session, alternating 30 mins aerobic and 30 mins resistance training		
		AE	20	16.28 ± 1.34	35.1 ± 0.9		3 times/week, intensity at ventilatory threshold I (VTI), 60 mins/session,		

								pure aerobic for first 14 weeks, aerobic + light resistance for weeks 14-26		
Lee et al.	2010	CE	20		12-14	26.12 ± 2.44	Overweight	3 times/week, 60 mins/session, including 2 sets circuit weight training (70-80% max strength) and 1 set aerobic exercise (60-80% VO2max or 70-90% HRmax)	BIA	WC, BMI, TC, TG, HDL-C, LDL-C
		AE	16		12-14	26.34 ± 2.25		3 times/week, 60 mins/session, various aerobic sports, intensity controlled at 60-80% VO2max or 70-90% HRmax		
Lee et al.	2019	CE	40	14/26	14.5 ± 1.7	32.3 ± 4.1	Obesity	3 times/week, intensity 50-65% Peak VO2 and 12-15 reps (to exhaustion), 60 mins/session, including 30 mins aerobic and 30 mins resistance training	DXA	LBM, WC, BMI, BW
		AE	38	13/25	14.4 ± 1.6	33.7 ± 4.0		3 times/week, intensity 50-65% Peak VO2, 60 mins/session, treadmill or elliptical training		
Lee et al.	2020	CE	40	14/26	14.5 ± 1.7	32.3 ± 4.1	Obesity	3 times/week, intensity 50-65% Peak VO2 and 12-15 reps (to exhaustion), 60 mins/session, including 30 mins aerobic and 30 mins resistance training	DXA	BW, TC, HDL-C, LDL-C, TG, SBP, DBP
		AE	38	13/25	14.4 ± 1.6	33.7 ± 4.0		3 times/week, intensity 50-65% Peak VO2, 60 mins/session, treadmill or elliptical training		
Loria-Kohen et al.	2013	CE	22	10/12	36.71 ± 6.99	28.32 ± 1.54	Overweight	3 times/week, intensity 50-60% 15RM and HRR, avg. 67 mins/session, circuit training interleaving aerobic machines and resistance exercises	DXA	BMI, WC, LBM
		AE	25	10/15	35.69 ± 8.07	28.91 ± 1.78		3 times/week, intensity 50-60% HRR, avg. 67 mins/session, bike or elliptical		

								training		
Monteiro et al.	2015	CE	14	9/5	11.03 ± 1.34	33.17 ± 4.7	Obesity	3 times/week, intensity 55-75% RM and 65-85% VO2peak, 60 mins/session, first 30 mins resistance circuit training then 30 mins aerobic (run/walk) training	DXA	BW, BMI, BFP, LBM, WC, TC, TG, HDL-C, LDL-C
		AE	18	10/8	11.00 ± 1.02	30.15 ± 2.90		3 times/week, intensity 65-85% VO2peak, 50 mins/session, walking and running training		
Park et al.	2003	CE	10	0/10	43.4 ± 1.04	25.8 ± 1.43	Overweight	6 times/week, intensity 60-70% HRmax and 60-70% 1RM, 60 mins/session, alternating aerobic and resistance training every other day	BIA	LBM, BW, BFP, TC, TG, HDL-C, LDL-C
		AE	10	0/10	42.2 ± 1.91	25.3 ± 1.74		6 times/week, intensity 60-70% HRmax, 60 mins/session, aerobics or brisk walking training		
Ramezani et al.	2017	CE	15	15/0	10.05 ± 1.41	30-35	Obesity	4 times/week, intensity 50-75% HRR and 50-75% 1RM, approx. 58 mins/session, 2 running and 2 circuit resistance training sessions performed on different days	NR	TC, TG, LDL-C, HDL-C, BMI
		AE	15	15/0	10.05 ± 1.41	30-35		4 times/week, intensity 50-75% HRR, approx. 58 mins/session (incl. intervals), interval running training (4-7 sets x 5 mins)		
Rejeki et al.	2023	CE	10	0/10	22.00 ± 0.94	30.76 ± 3.53	Obesity	3 times/week, intensity 60-70% HRmax and 60-70% 1RM, approx. 45 mins/session, aerobic or resistance training performed on separate days	BIA	BW, BMI

		AE	10	0/10	21.60 ± 1.65	30.82 ± 3.55		3 times/week, intensity 60-70% HRmax, 45 mins/session, treadmill running training	BIA	
Said et al.	2021	CE	14	14/0	21.74 ± 1.42	36.11 ± 1.47	Obesity	4 times/week, intensity 50-70% HRmax and 50-55% 1RM (increasing monthly), 60 mins/session, including 30 mins aerobic and 30 mins resistance training	BIA	BW, BMI, WC, BFP, BP, TG, TC, HDL-C, LDL-C
		AE	13	13/0	21.74 ± 1.42	36.3 ± 2.52		4 times/week, intensity 50-70% HRmax, 60 mins/session, treadmill, bike, or elliptical training		
Sanal et al.	2013	CE	32	16/16	39.0 ± 9.7	31.9 ± 4.0	Obesity	3-5 times aerobic and 2 times resistance/week, intensity 50-85% HRmax and 50-80% 1RM, cycle ergometer and 6-station resistance training	DXA	BW, BMI, WC, BFP
		AE	33	15/18	39.0 ± 10.5	31.4 ± 4.8		3-5 times/week, intensity 50-85% HRmax, avg. 25 mins/session (progressive), cycle ergometer training		
Sigal et al.	2014	CE	75	22/53	15.5 ± 1.3	34.7 ± 4.3	Obesity	4 times/week, intensity 65-85% HRmax and 8-RM (approx. 80% 1RM), performing complete aerobic training (20-45 mins) and resistance training (7 exercises) each session	MRI	BW, BFP, LBM, WC, BMI, SBP, DBP
		AE	75	22/53	15.5 ± 1.4	34.7 ± 4.3		4 times/week, intensity 65-85% HRmax, avg. 33 mins/session (progressively 20-45 mins), treadmill, elliptical, or bike training		
Tseng et al.	2013	CE	10	10/0	22.2 ± 2.21	31.2 ± 3.79	Obesity	5 times/week, intensity 50-70% HRmax and 50-80% 1RM, 60 mins/session, alternating aerobic and	NR	HDL-C, TG, SBP, DBP, BW, BMI, WC

								resistance training every other day		
		AE	10	10/0	22.1 ± 1.89	31.0 ± 3.48		5 times/week, intensity 50-70% HRmax, 60 mins/session, brisk walking on treadmill		
Thomson et al.	2008	CE	33	0/33	29.3 ± 6.8	36.1 ± 4.8	Obesity	5 times/week (3 aerobic + 2 resistance), intensity 60-80% HRmax and 50-75% 1RM, 25-45 mins aerobic or 3 sets of 12 reps resistance per session, performed on different days	DXA	BW, BFP, WC, SBP, DBP, TG, TC, LDL-C, HDL-C, INS, HOMA-IR
		AE	31	0/31	29.3 ± 6.8	36.1 ± 4.8		5 times/week, intensity 60-80% HRmax, 25-45 mins/session (progressive), walking or jogging training		
Wang et al.	2017	CE	12	NR	NR	29.43 ± 3.65	Obesity	3 times/week, intensity at 60-70% HRmax and 60-70% 1RM, 60 min/session, including 40 min aerobic and 20 min resistance training	NR	BW, BMI, BFP, TG, TC, LDL-C, HDL-C
		AE	12	NR	NR	31.09 ± 3.98		3 times/week, intensity at 60-70% HRmax, 60 min/session, aerobics and jogging		
Wang et al.	2025	CE	25	0/25	27.76 ± 4.00	30.35 ± 1.67	Obesity	3 times/week, intensity 40-59% VO2peak and 60% 1RM, 60 mins/session, alternating aerobic and resistance circuit training (5 circuits)	NR	INS, HOMA-IR, SBP, DBP
		AE	21	0/21	29.43 ± 4.32	29.75 ± 1.78	Overweight	3 times/week, intensity 40-59% VO2peak, 60 mins/session, treadmill, elliptical, recumbent bike, or rowing machine training		

Note: Outcome Indicators: BW = Body Weight; BMI = Body Mass Index; BFP = Body Fat Percentage; LBM = Lean Body Mass; WC = Waist Circumference; SBP = Systolic Blood Pressure;

DBP = Diastolic Blood Pressure; BP = Blood Pressure; TC = Total Cholesterol; TG = Triglycerides; HDL-C = High-Density Lipoprotein Cholesterol; LDL-C = Low-Density Lipoprotein Cholesterol; INS = Insulin; HOMA-IR = Homeostatic Model Assessment for Insulin Resistance. Intervention Measures: HRmax = Maximum Heart Rate; HRR = Heart Rate Reserve; RM = Repetition Maximum; RPE = Rating of Perceived Exertion; VO_2max = Maximum Oxygen Uptake; VO_2peak = Peak Oxygen Uptake; VT/VTI = Ventilatory Threshold. Body Composition Methods: DXA = Dual-Energy X-ray Absorptiometry; BIA = Bioelectrical Impedance Analysis; ADP = Air Displacement Plethysmography; MRI = Magnetic Resonance Imaging; NR = Not Reported.

Supplemental Table 2. Detailed Table of Exercise Interventions.

Author	Year	Group	Duration (Weeks)	CT Modality Order	ning Frequency(sessions/week)	Avg Session Duration(min)	Total Weekly Duration(min)	Training Intensity Monitoring Indicators
Ambelu et al.	2023	CE	12	NR	3	60	180	40-70% HRR; RPE 11-13
Ambelu et al.	2023	AE	12	N/A	3	60	180	40-70% HRR
Ackel-D’Elia et al.	2014	CE	24	Alternating	3	60	180	Aerobic: VT1 ± 4bpm; Resistance: 6-20RM
Ackel-D’Elia et al.	2014	AE	24	N/A	3	60	180	VT1 ± 4bpm
Alemayehu et al.	2023	CE	12	NR	NR	60	NR	64-76% HRmax; RPE 11-13
Alemayehu et al.	2023	AE	12	N/A	NR	60	NR	64-76% HRmax
Benito et al.	2020	CE	22	Alternating	3	55	165	50-60% HRR; 50-60% 15RM
Benito et al.	2020	AE		N/A	3	55	165	50-60% HRR
Campos et al.	2014	CE	52	NR	3	60	180	Aerobic: VT1 ± 4bpm; Resistance: 6-20RM
Campos et al.	2014	AE	52	N/A	3	60	180	VT1 ± 4bpm
Carnier et al.	2013	CE	52	Aerobic then Resistance	3	60	180	Aerobic: VT1 ± 4bpm; Resistance: 6-20RM
Carnier et al.	2013	AE	52	N/A	3	60	180	VT1 ± 4bpm
Castro et al.	2020	CE	22	NR	3	56.5	169.5	Aerobic: 50-60% HRR; Resistance: 15RM

Castro et al.	2020	AE	22	N/A	3	56.5	169.5	50-60% HRR
Chen et al.	2015	CE	8	Resistance then Aerobic	3	60	180	60% VO2max; 8-12RM
Chen et al.	2015	AE	8	N/A	3	60	180	60% VO2max
Choo et al.	2014	CE	39	Resistance then Aerobic	3	60	180	50-70% HRR; 8-12 Reps (approx 40-60% max strength)
Choo et al.	2014	AE	39	N/A	3	60	180	50-70% HRR
Dâmaso et al.	2014	CE	52	Alternating	3	60	180	VT I (~50-70% VO2peak); 1RM (to exhaustion)
Dâmaso et al.	2014	AE	52	N/A	3	60	180	VT I (~50-70% VO2peak)
Davis et al.	2024	CE	12	NR	3	50	150	40-80% HRR; 40-80% 1RM
Davis et al.	2024	AE	12	N/A	3	50	150	40-80% HRR
de Mello et al.	2011	CE	52	Alternating	3	60	180	VT I (~50-70% VO2peak); RM (6-20RM)
de Mello et al.	2011	AE	52	N/A	3	60	180	VT I (~50-70% VO2peak)
de Piano et al.	2012	CE	52	Alternating	3	60	180	VT I (~50-70% VO2peak); Reps (6-20)
de Piano et al.	2012	AE	52	N/A	3	60	180	VT I (~50-70% VO2peak)
Inoue et al.	2015	CE	52	Alternating	3	60	180	VT I (VTI); RM (15-20, 10-12, 6-8RM)
Inoue et al.	2015	CE	52	Alternating	3	60	180	VT I (VTI); RM (15-20, 10-12, 6-8RM)
Inoue et al.	2015	AE	52	N/A	3	60	180	VT I (VTI)
Lee et al.	2010	CE	10	Alternating	3	60	180	60-80% VO2max, 70-90% HRmax; 70-80% max strength
Lee et al.	2010	AE	10	N/A	3	60	180	60-80% VO2max, 70-90% HRmax
Lee et al.	2019	CE	24	Aerobic then Resistance	3	60	180	50-65% Peak VO2; 12-15 Reps (to exhaustion)
Lee et al.	2019	AE	24	N/A	3	60	180	50-65% Peak VO2
Lee et al.	2020	CE	24	Aerobic then Resistance	3	60	180	50-65% Peak VO2; 12-15 Reps (to exhaustion)
Lee et al.	2020	AE	24	N/A	3	60	180	50-65% Peak VO2
Loria-Kohen et al.	2013	CE	22	Alternating	3	67	201	50-60% HRR; 50-60% 15RM

Loria-Kohen et al.	2013	AE	22	N/A	3	67	201	50-60% HRR
Makiel et al.	2023	CE	12	Resistance then Aerobic	3	60	180	50-70% HRmax; 50-70% 1RM
Makiel et al.	2023	AE	12	N/A	3	60	180	70% HRmax
Monteiro et al.	2015	CE	20	Resistance then Aerobic	3	60	180	65-85% VO2peak; 55-75% RM
Monteiro et al.	2015	AE	20	N/A	3	50	150	65-85% VO2peak
Park et al.	2003	CE	24	Alternating	6	60	360	60-70% HRmax; 60-70% 1RM
Park et al.	2003	AE	24	N/A	6	60	360	60-70% HRmax
Piano et al.	2012	CE	52	Alternating	3	60	180	Aerobic: VT I; Resistance: 6-20 Reps
Piano et al.	2012	AE	52	NA	3	60	180	VT I (50-70% VO2peak)
Ramezani et al.	2017	CE	8	NR	4	58	232	50-75% HRR; 50-75% 1RM
Ramezani et al.	2017	AE	8	N/A	4	58	232	50-75% HRR
Rejeki et al.	2023	CE	4	Alternating	3	NR	NR	60-70% HRmax; 60-70% 1RM
Rejeki et al.	2023	AE	4	N/A	3	45	135	60-70% HRmax
Said et al.	2021	CE	12	Aerobic then Resistance	4	60	240	50-70% HRmax; 50-80% 1RM
Said et al.	2021	AE	12	N/A	4	60	240	50-70% HRmax
Sanal et al.	2013	CE	12	NR	N/A	NR	NR	50-85% HRmax; 50-80% 1RM
Sanal et al.	2013	AE	12	N/A	N/A	NR	NR	50-85% HRmax
Sigal et al.	2014	CE	22	NR	4	NR	NR	65-85% HRmax; 8-RM
Sigal et al.	2014	AE	22	N/A	4	33	132	65-85% HRmax
Tseng et al.	2013	CE	12	Alternating	5	60	300	50-70% HRmax; 50-80% 1RM
Tseng et al.	2013	AE	12	N/A	5	60	300	50-70% HRmax
Thomson et al.	2008	CE	20	NR	5	NR	NR	60-80% HRmax; 50-75% 1RM
Thomson et al.	2008	AE	20	N/A	5	35	175	60-80% HRmax
Wang	2017	CE	16	Aerobic then Resistance	3	60	180	60-70% HRmax; 60-70% 1RM

Wang	2017	AE	16	N/A	3	60	180	60-70% HRmax
Wang et al.	2025	CE	20	Alternating	3	60	180	40-59% VO2peak; 60% 1RM
Wang et al.	2025	AE	20	N/A	3	60	180	40-59% VO2peak

Note: NR: signifies Not Reported, N/A: signifies Not Applicable, VT: signifies Ventilatory Threshold

Supplemental Table 3. Subgroup analysis results for BW.

Subgroup	K(n)	MD	95% CI	P_d	Q	I^2/%	P_m
**Age**							0.33
Children and adolescents	13(682)	1.76	[-0.57, 4.09]	0.14	31.30	64	
Adults	11(470)	0.30	[-1.54, 2.13]	0.75	12.83	33	
**BMI**							0.06
Overweight	6(210)	-0.69	[-2.87, 1.49]	0.53	7.47	38	
Obesity	19(966)	1.99	[0.27, 3.72]	0.02	36.63	47	
**Intervention period**							<0.01
≤ 12 weeks	7(213)	-1.79	[-3.30, -0.28]	0.02	3.66	0	
> 12 weeks	18(963)	2.35	[0.80, 3.90]	<0.01	29.49	38	
**Training frequency**							0.82
≤ 3 times/week	18(845)	1.35	[-0.48, 3.18]	0.15	43.66	57	
> 3 times/week	5(243)	1.70	[-0.69, 4.09]	0.16	1.47	0	
**Average training duration per session**							0.34
< 60 minutes	3(165)	-0.35	[-3.22, 2.52]	0.81	2.34	18	
≥ 60 minutes	18(1011)	1.33	[-0.52, 3.18]	0.16	57.56	63	
**Weekly total training duration**							0.20
< 180 minutes	3(165)	-0.35	[-3.22, 2.52]	0.81	2.34	18	
≥ 180 minutes	17(727)	1.86	[0.06, 3.66]	0.04	33.03	51	
**Training order**							0.02
Aerobic before resistance	4(203)	4.09	[2.38, 5.80]	<0.01	4.41	23	
Resistance before aerobic	3(128)	3.90	[-5.23, 13.02]	0.40	12.01	85	
Alternately	10(396)	0.53	[-1.34, 2.41]	0.58	5.85	0	
**Intensity**							0.13
Moderate-intensity	11(399)	-0.10	[-2.06, 1.86]	0.92	13.19	31	
High-intensity	14(777)	2.06	[0.05, 4.08]	0.04	33.87	59	

Note: K(n), number of included studies (total pooled sample size); MD, effect size (mean difference); 95% CI, 95% confidence interval; P_d, P-value of the pooled effect size; P_m, P-value for subgroup differences.

Supplemental Table 4. Subgroup analysis results for BMI.

Subgroup	K(n)	MD	95% CI	P_d	Q	I^2/%	P_m
**Age**							**<0.01**
Children and adolescents	14(718)	0.51	[-0.16, 1.19]	0.14	22.90	45.96	
Adults	10(376)	-0.73	[-1.21, -0.26]	0.00	8.96	12.40	
**BMI**							**0.02**

Overweight	7(223)	-0.84	[-1.52, -0.16]	0.02	9.83	35.00	
Obesity	18(1048)	0.22	[-0.33, 0.78]	0.43	33.80	46.42	
**Intervention period**							**0.93**
≤ 12 weeks	10(376)	-0.16	[-0.95, 0.63]	0.69	19.32	52.77	
> 12 weeks	15(895)	-0.11	[-0.78, 0.56]	0.74	47.25	62.79	
**Training frequency**							**0.63**
≤ 3 times/week	19(1044)	-0.10	[-0.67, 0.47]	0.73	54.13	57.37	
> 3 times/week	4(227)	0.21	[-0.92, 1.34]	0.72	4.95	43.19	
**Average training duration per session**							**0.74**
< 60 minutes	4(191)	0.01	[-0.97, 0.99]	0.99	8.20	64.29	
≥ 60 minutes	18(1080)	-0.19	[-0.87, 0.49]	0.58	61.83	65.24	
**Weekly total training duration**							**0.16**
< 180 minutes	3(127)	-0.48	[-1.11, 0.16]	0.14	1.58	0.00	
≥ 180 minutes	17(1022)	0.18	[-0.49, 0.85]	0.60	39.90	59.59	
**Training order**							**0.06**
Aerobic before resistance	3(155)	-0.06	[-1.53, 1.40]	0.93	9.06	75.65	
Resistance before aerobic	2(62)	2.29	[-0.10, 4.68]	0.06	2.11	52.65	
Alternately	11(521)	-0.57	[-1.11, -0.04]	0.04	8.95	0.00	
**Intensity**							**0.01**
Moderate-intensity	11(474)	-0.81	[-1.43, -0.19]	0.01	13.81	29.53	
High-intensity	13(797)	0.25	[-0.32, 0.82]	0.40	21.44	44.24	

Supplemental Table 5. Subgroup analysis results for BFP.

**Subgroup**	**K(n)**	**MD**	**95% CI**	**P_d**	**Q**	**I^2/%**	**P_m**
**Age**							0.05
Children and adolescents	6(391)	0.82	[-0.40, 2.05]	0.19	2.22	0.00	
Adults	8(337)	-0.64	[-1.44, 0.16]	0.12	6.99	0.00	
**BMI**							0.29
Overweight	5(117)	-0.89	[-2.24, 0.46]	0.20	3.93	0.00	
Obesity	10(635)	-0.02	[-0.90, 0.86]	0.96	9.33	20.62	
**Intervention period**							0.14

≤ 12 weeks	6(193)	-0.78	[-1.65, 0.09]	0.08	3.86	0.00	
> 12 weeks	9(559)	0.24	[-0.80, 1.27]	0.66	7.89	11.15	
**Training frequency**							0.84
≤ 3 times/week	9(405)	-0.20	[-1.36, 0.96]	0.73	9.51	25.72	
> 3 times/week	4(259)	-0.04	[-1.16, 1.08]	0.95	3.63	20.24	
**Average training duration per session**							0.21
< 60 minutes	2(118)	-1.42	[-2.89, 0.05]	0.06	0.00	0.00	
≥ 60 minutes	10(357)	-0.33	[-1.20, 0.55]	0.46	9.18	3.39	
**Weekly total training duration**							0.25
< 180 minutes	2(118)	-1.42	[-2.89, 0.05]	0.06	0.00	0.00	
≥ 180 minutes	8(314)	-0.41	[-1.30, 0.48]	0.37	6.27	0.00	
**Training order**							0.44
Aerobic before resistance	2(51)	-0.91	[-2.03, 0.21]	0.11	0.34	0.00	
Resistance before aerobic	2(62)	0.70	[-2.04, 3.43]	0.62	1.82	45.00	
Alternately	4(201)	0.18	[-1.94, 2.31]	0.87	1.90	0.00	
**Intensity**							0.04
Moderate-intensity	4(157)	-1.64	[-3.12, -0.15]	0.03	0.06	0.00	
High-intensity	11(595)	0.16	[-0.71, 1.03]	0.72	10.36	22.13	

Supplemental Table 6. Subgroup analysis results for waist circumference.

Subgroup	K(n)	MD	95% CI	P_d	Q	I^2/%	P_m
**Age**							0.51
Children and adolescents	5(288)	0.75	[-3.26, 4.75]	0.71	13.79	82.64	
Adults	8(364)	-0.74	[-2.51, 1.04]	0.41	8.05	17.62	
**BMI**							0.27
Overweight	4(196)	-1.17	[-3.56, 1.22]	0.34	3.20	3.62	
Obesity	9(456)	0.69	[-1.63, 3.00]	0.56	23.05	71.94	
**Intervention period**							0.22
≤ 12 weeks	5(176)	-1.34	[-3.28, 0.60]	0.17	2.20	0.00	
> 12 weeks	8(476)	0.74	[-1.98, 3.47]	0.59	20.91	76.66	

**Training frequency**							0.84
≤ 3 times/week	8(364)	-0.11	[-2.88, 2.65]	0.94	28.21	80.84	
> 3 times/week	4(223)	0.26	[-2.27, 2.80]	0.84	1.84	0.00	
**Average training duration per session**							0.06
< 60 minutes	2(75)	-2.60	[-4.90, -0.31]	0.03	0.02	0.00	
≥ 60 minutes	8(336)	0.74	[-1.91, 3.38]	0.59	16.72	67.68	
**Weekly total training duration**							0.06
< 180 minutes	2(75)	-2.60	[-4.90, -0.31]	0.03	0.02	0.00	
≥ 180 minutes	8(336)	0.74	[-1.91, 3.38]	0.59	16.72	67.68	
**Training order**							< 0.01
Aerobic before resistance	2(105)	1.58	[1.03, 2.14]	0.00	0.36	0.00	
Resistance before aerobic	2(98)	5.49	[0.42, 10.57]	0.03	2.16	53.60	
Alternately	5(180)	-2.25	[-4.56, 0.06]	0.06	1.95	0.00	
**Intensity**							0.02
Moderate-intensity	3(124)	-3.01	[-5.80, -0.23]	0.03	0.87	0.00	
High-intensity	10(528)	1.05	[-0.88, 2.98]	0.28	20.37	63.06	

Supplemental Table 7. Subgroup analysis results for LBM.

Subgroup	K(N)	Hedges' g	95% CI	P_d	Q	I^2/%	P_m
**Age**							0.06
Children and adolescents	11(614)	0.75	0.43~1.07	<0.01	40.02	71.01	
Adults	5(232)	0.35	0.08~0.62	0.01	6.12	6.82	
**BMI**							0.77
Overweight	3(114)	0.56	-0.09~1.22	0.09	5.39	65.03	
Obesity	13(732)	0.67	0.38~0.96	<0.01	44.27	70.49	
**Intervention period**							0.27
≤ 12 weeks	1(28)	0.24	-0.48~0.96	0.52	0.00	0	
> 12 weeks	15(818)	0.67	0.40~0.94	<0.01	49.19	69.66	

**Training frequency**							0.88
≤ 3 times/week	14(678)	0.68	0.42~0.93	<0.01	33.70	61.35	
> 3 times/week	2(168)	0.58	-0.70~1.85	0.37	6.78	85.26	
**Average training duration per session**							0.03
< 60 minutes	3(165)	0.33	0.03~0.63	0.03	0.93	0.00	
≥ 60 minutes	12(533)	0.81	0.52~1.10	<0.01	27.26	60.44	
**Weekly total training duration**							0.03
< 180 minutes	3(165)	0.33	0.03~0.63	0.03	0.93	0.00	
≥ 180 minutes	12(533)	0.81	0.52~1.10	<0.01	27.26	60.44	
**Training order**							0.01
Aerobic before resistance	1(78)	1.42	0.92~1.91	N/A	N/A	N/A	
Resistance before aerobic	1(32)	0.15	-0.53~0.83	N/A	N/A	N/A	
Alternately	9(403)	0.80	0.48~1.13	<0.01	17.75	56.70	
**Intensity**							0.64
Moderate-intensity	9(379)	0.71	0.35~1.06	<0.01	21.59	63.49	
High-intensity	7(467)	0.58	0.18~0.98	<0.01	28.02	74.95	

Note: K(N): number of included studies (total pooled sample size); Hedges' g: Combined effect size; P_d: P-value for the subgroup effect size; P_m: P-value for the difference between subgroups (Test of group differences); Q & I^2: Heterogeneity statistics; N/A: Not applicable.

Secondary cardiometabolic outcomes

SBP

The meta-analysis, incorporating 10 independent studies, showed no statistically significant between-group difference between CE and AE in post-intervention systolic blood pressure among adolescents and adults with overweight or obesity (MD = -2.19 mmHg, 95% CI: -4.59 to 0.21, P = 0.07). Between-study heterogeneity was moderate (τ^2 = 7.98, I^2 = 59.93%, H^2 = 2.50; Q(9) = 24.49, P < 0.001) (Figure. 1).

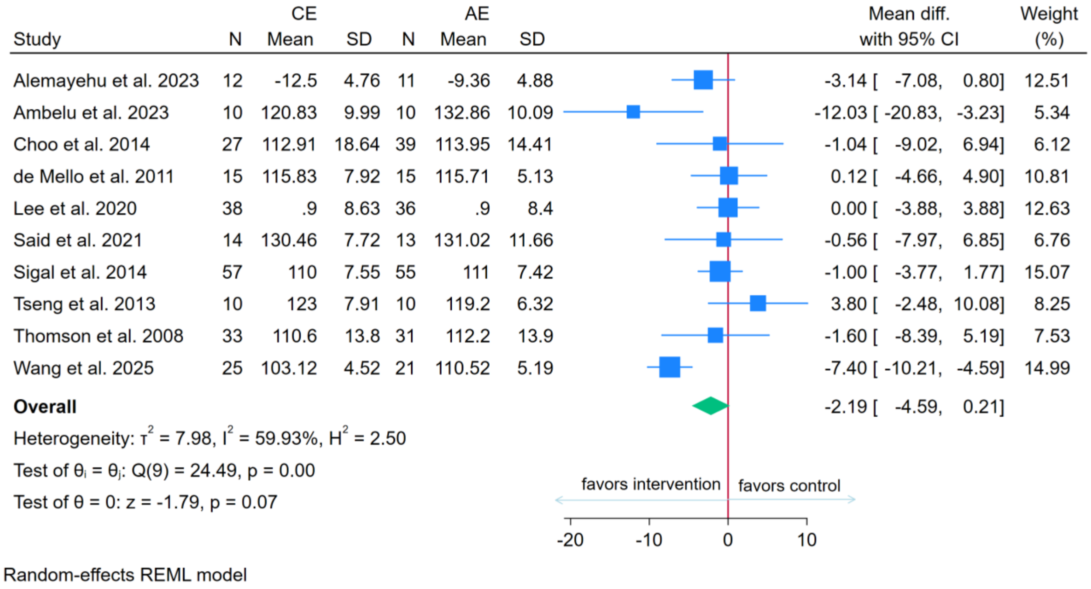

Supplemental Figure 1. Meta-analysis results for SBP.

Subgroup analysis for SBP (Table 8) revealed that no significant subgroup effects were observed concerning age, BMI classification, intervention duration, training frequency, training sequence, or exercise intensity ($P > 0.05$).

Meta-regression analyses were conducted to evaluate the potential moderating effects of the following continuous variables on the pooled main effect: age ($\beta = -0.17$, 95% *CI*: -0.39 to 0.04, $P = 0.10$), baseline BMI ($\beta = 0.58$, 95% *CI*: -0.17 to 1.33, $P = 0.13$), intervention duration ($\beta = 0.07$, 95% *CI*: -0.13 to 0.28, $P = 0.47$), training frequency ($\beta = 2.47$, 95% *CI*: -0.85 to 5.80, $P = 0.15$), and total weekly training duration ($\beta = 0.06$, 95% *CI*: -0.02 to 0.14, $P = 0.14$). The results demonstrated that none of the aforementioned continuous variables exerted a statistically significant moderating effect on the differential efficacy between CE and AE in reducing SBP ($P > 0.05$).

Supplemental Table 8. Subgroup analysis results for SBP.

Subgroup	K(N)	MD	95% CI	P_d	Q	I^2 /%	P_m
**Age**							0.19
Children and adolescents	3(216)	-0.52	-2.56~1.52	0.62	0.25	0.00	
Adults	7(266)	-3.23	-6.73~0.28	0.07	16.60	62.88	
**BMI**							0.28
Overweight	3(109)	-4.68	-9.96~0.60	0.08	3.91	47.15	
Obesity	7(373)	-1.40	-4.26~1.45	0.34	19.75	65.84	
**Intervention period**							0.91

Subgroup	K(N)	MD	95% CI	P_d	Q	I^2/%	P_m
≤ 12 weeks	4(90)	-2.53	-8.31~3.25	0.39	8.71	69.94	
> 12 weeks	6(392)	-2.14	-5.03~0.75	0.15	15.68	64.08	
**Training frequency**							0.19
≤ 3 times/week	5(236)	-3.67	-7.98~0.63	0.09	16.28	73.71	
> 3 times/week	4(223)	-0.40	-2.66~1.86	0.73	2.02	0.00	
**Training order**							0.92
Aerobic before resistance	2(101)	-0.12	-3.56~3.32	0.95	0.02	0.00	
Resistance before aerobic	1(66)	-1.04	-9.02~6.94	N/A	N/A	N/A	
Alternately	3(96)	-1.61	-8.22~5.01	0.63	14.38	84.64	
**Intensity**							0.22
Moderate-intensity	3(99)	-3.81	-8.13~0.51	0.08	8.06	74.40	
High-intensity	7(383)	-0.86	-2.72~0.99	0.36	8.56	0.00	

DBP

The meta-analysis, incorporating 9 independent studies, sshowed a small statistically significant between-group difference in post-intervention DBP favoring CE over AE among adolescents and adults with overweight or obesity (MD = -2.02 mmHg, 95% CI: -3.92 to -0.12, P = 0.04). Given the small absolute effect size and low certainty of evidence, the clinical relevance of this DBP difference should be interpreted cautiously. Between-study heterogeneity was moderate (τ^2 = 4.12, I^2 = 53.16%, H^2 = 2.13; Q(8) = 17.23, P = 0.03) (Figure. 2).

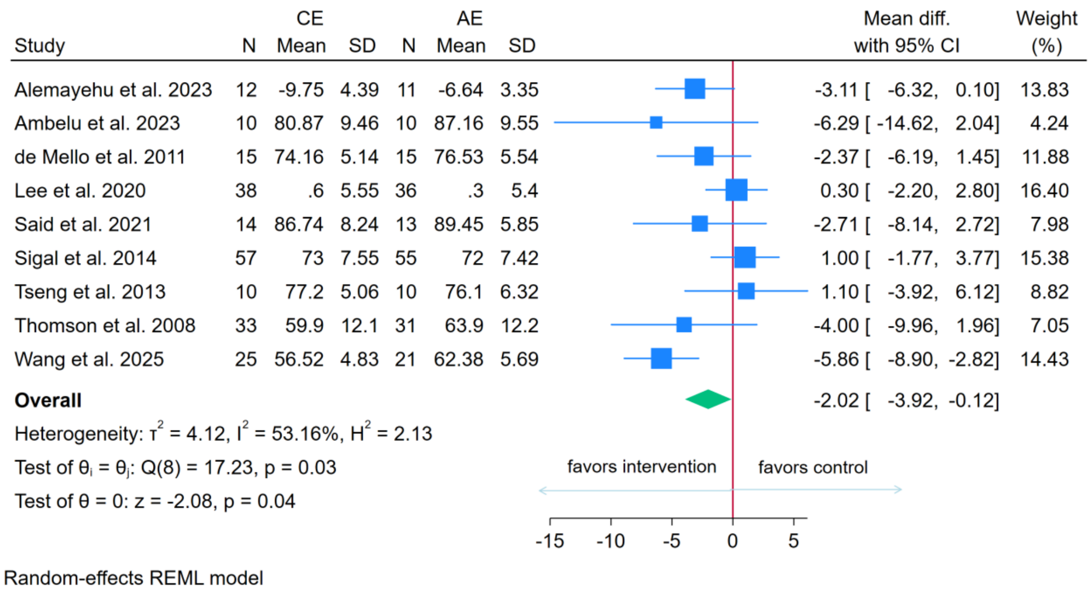

Supplemental Figure 2. Meta-analysis results for DBP.

Exploratory subgroup analysis for DBP (Table 9) suggested that the

DBP-lowering effect favoring CE was more evident in adult cohorts and moderate-intensity protocols. However, these findings should be interpreted cautiously because subgroup analyses were based on limited study numbers and were not adjusted for multiplicity. No significant subgroup effects were observed concerning BMI classification, intervention duration, training frequency, or training sequence (P > 0.05).

Table 9. Subgroup analysis results for DBP.

Subgroup	K(N)	MD	95% CI	P_d	Q	I^2/%	P_m
**Age**							0.01
Children and adolescents	3(216)	0.04	-1.62~1.71	0.96	2.03	0.00	
Adults	6(200)	-3.53	-5.68~-1.38	0.00	6.09	24.96	
**BMI**							0.32
Overweight	2(43)	-3.52	-6.52~-0.52	0.02	0.49	0.00	
Obesity	7(373)	-1.63	-3.84~0.58	0.15	15.08	59.08	
**Intervention period**							0.80
≤ 12 weeks	4(90)	-2.38	-4.71~-0.05	0.04	2.91	0.00	
> 12 weeks	5(326)	-1.92	-4.66~0.82	0.17	13.90	70.13	
**Training frequency**							0.19
≤ 3 times/week	4(170)	-2.99	-6.29~0.32	0.08	10.31	67.73	
> 3 times/week	4(223)	-0.29	-2.60~2.02	0.81	3.36	12.22	
**Training order**							0.27
Aerobic before resistance	2(101)	-0.23	-2.49~2.04	0.85	0.97	0.00	
Alternately	3(96)	-2.77	-6.64~1.10	0.16	5.88	66.22	
**Intensity**							0.01
Moderate-intensity	3(99)	-3.97	-6.12~-1.81	0.00	2.43	20.47	
High-intensity	6(317)	-0.18	-1.75~1.39	0.82	5.57	0.00	

Meta-regression analyses were conducted to evaluate the potential moderating effects of the following continuous variables on the pooled main effect: age (β = -0.16, 95% *CI*: -0.32 to 0.00, *P* = 0.05), baseline BMI (β = 0.24, 95% *CI*: -0.43 to 0.91, *P* = 0.48), intervention duration (β = 0.01, 95% *CI*: -0.15 to 0.18, *P* = 0.87), training frequency (β = 1.13, 95% *CI*: -1.68 to 3.93, *P* = 0.43), and total weekly training duration (β = 0.03, 95% *CI*: -0.03 to 0.09, *P* = 0.33). The results demonstrated that none of the aforementioned continuous variables exerted a statistically significant moderating effect on the differential efficacy between CE and AE in reducing DBP (*P* ≥ 0.05).

Insulin

The meta-analysis, incorporating 7 independent studies, showed that CE was associated with a statistically significant reduction in post-intervention insulin levels

compared with AE (MD = -2.75 uU/mL, 95% CI: -4.66 to -0.84, P < 0.01). Because this outcome was based on a limited number of studies and low-certainty evidence, the finding should be interpreted as suggestive rather than definitive. Between-study heterogeneity was moderate (τ^2 = 2.65, I^2 = 50.24%, H^2 = 2.01; Q(6) = 12.61, P = 0.05) (Figure. 3).

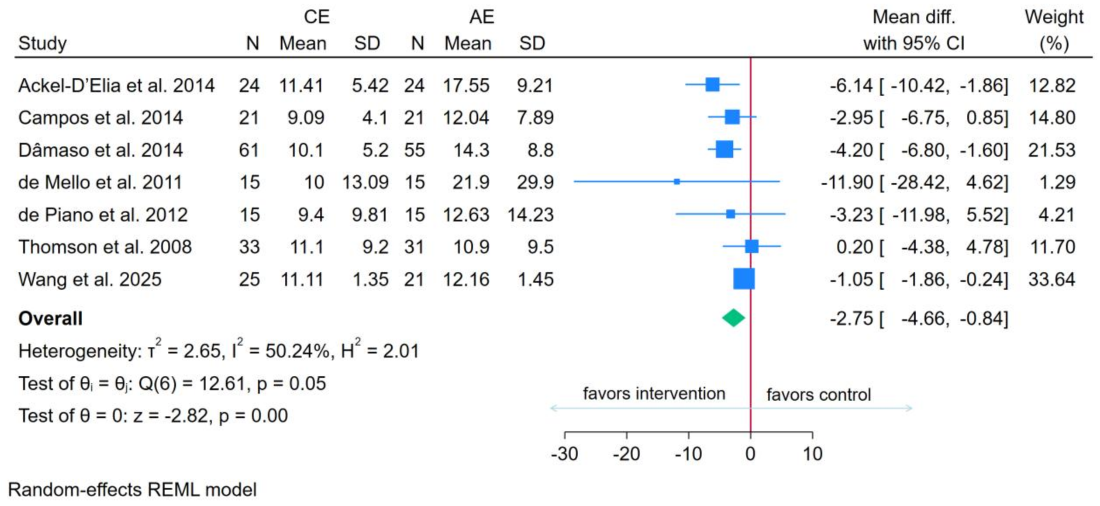

Supplemental Figure 3. Meta-analysis results for insulin.

Exploratory subgroup analysis for insulin (Table 10) suggested that the insulin-lowering effect favoring CE was observed in both children/adolescents and adults. No significant subgroup effects were observed concerning training frequency or exercise intensity (P > 0.05). Given the limited number of included studies, these subgroup findings should be considered hypothesis-generating.

Supplemental Table 10. Subgroup analysis results for insulin.

Subgroup	K(N)	MD	95% CI	P_d	Q	I^2/%	P_m
**Age**							＜0.01
Children and adolescents	5(266)	-4.32	-6.18~-2.46	0.00	2.07	0.00	
Adults	2(110)	-1.01	-1.81~-0.21	0.01	0.28	0.00	
**Training frequency**							0.19
≤ 3 times/week	6(312)	-3.20	-5.34~-1.07	0.00	12.05	54.96	
> 3 times/week	1(64)	0.20	-4.38~4.78	N/A	N/A	N/A	
**Intensity**							0.82
Moderate-intensity	5(196)	-3.01	-5.66~-0.36	0.03	7.77	49.69	
High-intensity	2(180)	-2.42	-6.65~1.81	0.26	2.68	62.68	

Meta-regression analyses were conducted to evaluate the potential moderating effects of the following continuous variables: age (β = 0.30, 95% *CI*: 0.12 to 0.48, *P* <

0.01), baseline BMI (β = -0.38, 95% *CI*: -0.84 to 0.07, *P* = 0.10), intervention duration (β = -0.08, 95% *CI*: -0.18 to 0.02, *P* = 0.11), and training frequency (β = 1.70, 95% *CI*: -1.36 to 4.76, *P* = 0.28). The results demonstrated that age exerted a statistically significant moderating effect on the differential efficacy between CE and AE in reducing insulin levels (Figure. 4); Specifically, the positive regression coefficient suggested that the relative insulin-lowering effect favoring CE tended to diminish with increasing age. This finding should be interpreted cautiously because the analysis was exploratory and based on a small number of studies. Baseline BMI, intervention duration, and training frequency did not demonstrate any significant moderating effects (*P* > 0.05).

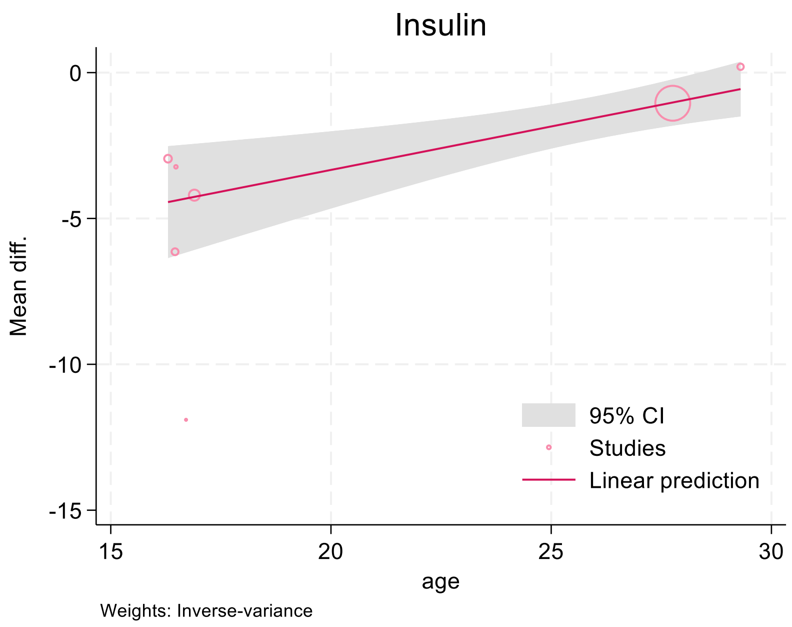

Supplemental Figure 4. Regression analysis results for insulin.

HOMA-IR

The meta-analysis, incorporating 7 independent studies, showed that CE was associated with a statistically significant reduction in HOMA-IR compared with AE (MD = -0.59, 95% CI: -1.00 to -0.18, P < 0.01). However, this result was based on a limited number of studies and low-certainty evidence; therefore, it should be interpreted cautiously. Moderate heterogeneity was observed across the included trials (I^2 = 55.37%, P = 0.07) (Figure. 5).

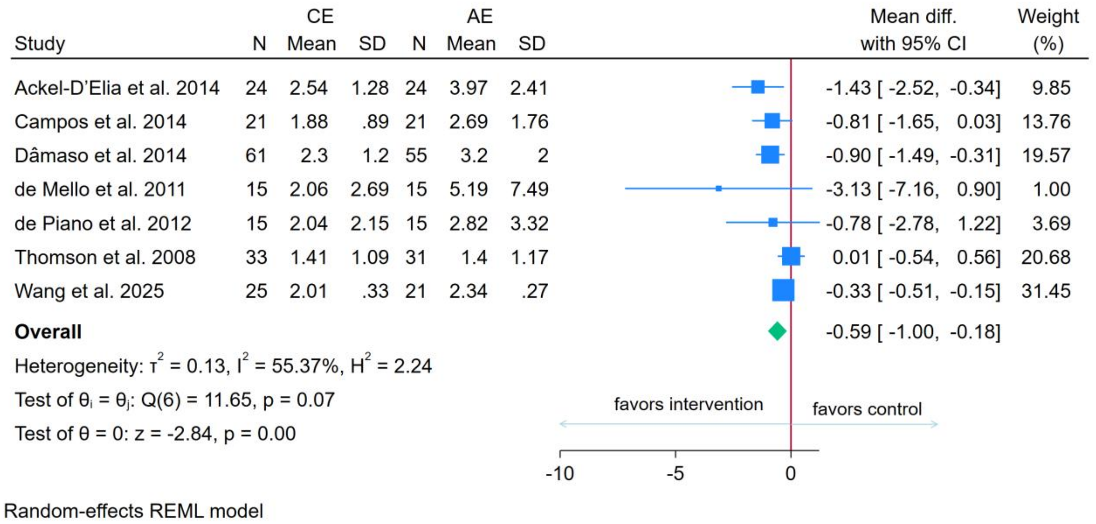

Supplemental Figure 5. Meta-analysis results for HOMA-IR.

Exploratory subgroup analysis for HOMA-IR (Supplementary Table 11) suggested that the reduction favoring CE was more evident in children/adolescents and in protocols with a training frequency ≤3 sessions per week. However, these subgroup findings should be interpreted cautiously because they were based on limited study numbers and were not adjusted for multiplicity. No significant subgroup effect was observed concerning exercise intensity (P > 0.05).

Supplemental Table 11. Subgroup analysis results for HOMA-IR.

Subgroup	K(N)	MD	95% CI	P_d	Q	I^2/%	P_m
**Age**							0.01
Children and adolescents	5(266)	-0.98	-1.41~-0.55	0.00	2.01	0.00	
Adults	2(110)	-0.27	-0.53~0.00	0.05	1.31	23.95	
**Training frequency**							0.04
≤ 3 times/week	6(312)	-0.74	-1.18~-0.29	0.00	9.48	46.54	
> 3 times/week	1(64)	0.01	-0.54~0.56	N/A	N/A	N/A	
**Intensity**							0.59
Moderate-intensity	5(196)	-0.73	-1.30~-0.16	0.01	6.80	43.40	
High-intensity	2(180)	-0.44	-1.33~0.45	0.34	4.83	79.29	

Meta-regression analyses were conducted to evaluate the potential moderating effects of the following continuous variables on the pooled main effect: age (β = 0.06, 95% *CI*: 0.02 to 0.10, P < 0.01), baseline BMI (β = -0.06, 95% *CI*: -0.23 to 0.10, P = 0.46), intervention duration (β = -0.02, 95% *CI*: -0.03 to 0.00, P = 0.02), and training

frequency (β = 0.37, 95% *CI*: -0.11 to 0.86, *P* = 0.13). The results demonstrated that age and intervention duration exerted statistically significant moderating effects on the differential efficacy between CE and AE in reducing HOMA-IR. Specifically, with advancing age, the superiority of CE over AE gradually diminished (Figure. 6); conversely, with prolonged intervention duration, the efficacy of CE in reducing HOMA-IR became significantly more pronounced compared to AE (Figure. 6). This finding should be interpreted cautiously because the analysis was exploratory and based on a small number of studies. Baseline BMI and training frequency did not demonstrate any significant moderating effects (*P* > 0.05).

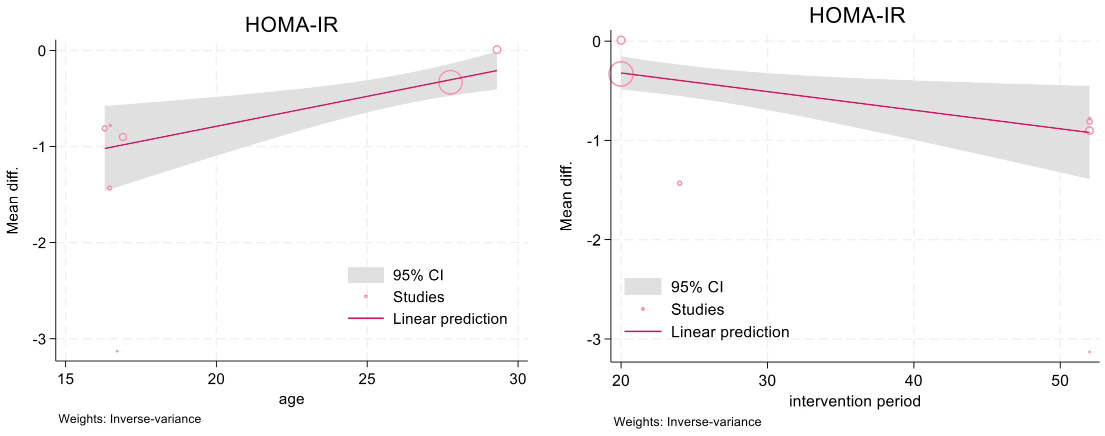

Supplemental Figure 6. Regression analysis results for HOMA-IR.

TC

The meta-analysis, incorporating 14 independent studies, showed no statistically significant between-group difference between CE and AE in post-intervention TC levels among adolescents and adults with overweight or obesity (MD = 0.04 mmol/L, 95% CI: −0.07 to 0.16, P = 0.46). Between-study heterogeneity was negligible (τ^2 = 0.00, I^2 = 0.00%, H^2 = 1.00; Q(13) = 6.65, P = 0.92) (Figure. 7).

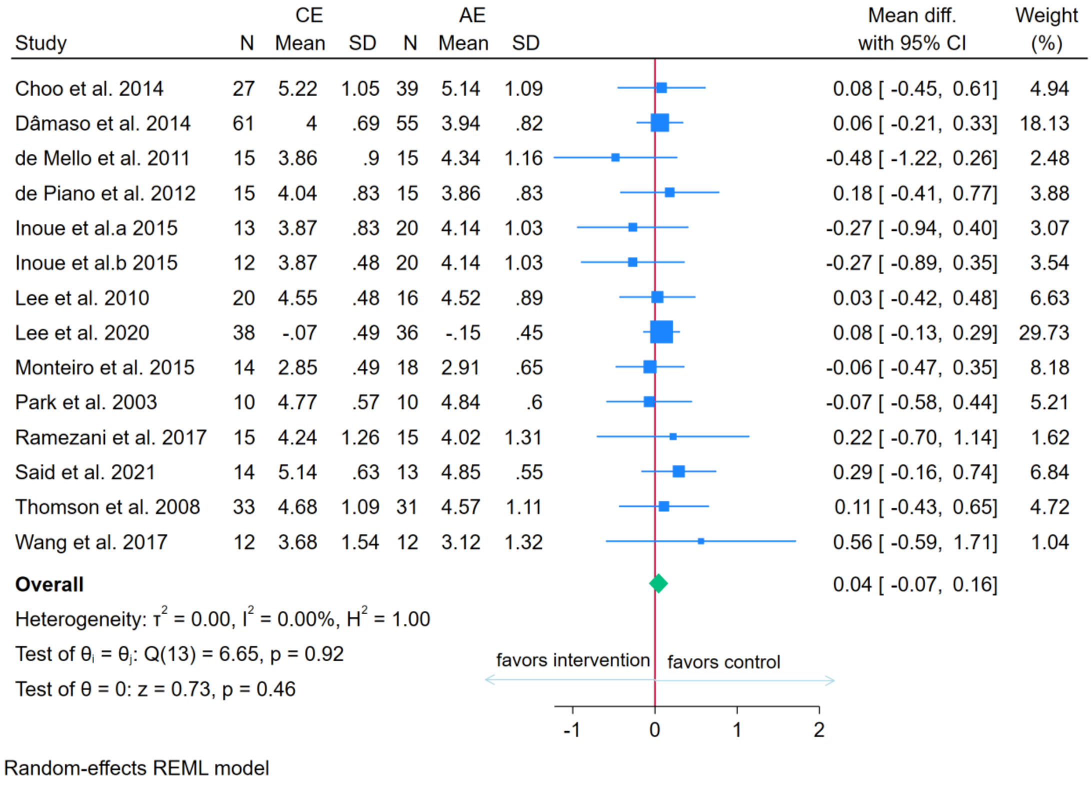

Supplemental Figure 7. Meta-analysis results for TC.

Subgroup analysis for TC (Supplementary Table 12) revealed that no significant subgroup effects were observed concerning age, BMI classification, intervention duration, training frequency, average training duration per session, training sequence, or exercise intensity ($P > 0.05$).

Supplemental Table 12. Subgroup analysis results for TC.

Subgroup	K(n)	MD	95% CI	P_d	Q	I^2/%	P_m
**Age**							0.51
Children and adolescents	9(413)	0.79	[-4.38, 5.95]	0.77	4.23	0	
Adults	4(177)	4.52	[-5.18, 14.22]	0.36	1.09	0	
**BMI**							0.99
Overweight	4(146)	1.89	[-8.81, 12.60]	0.73	0.96	0	
Obesity	10(468)	1.80	[-3.20, 6.81]	0.48	5.58	0	
**Intervention period**							0.37
≤ 12 weeks	3(93)	6.69	[-4.95, 18.33]	0.26	0.61	0	
> 12 weeks	11(521)	0.95	[-3.97, 5.87]	0.71	5.13	0	
**Training frequency**							0.48
≤ 3 times/week	10(473)	1.03	[-3.99, 6.06]	0.69	4.93	0	
> 3 times/week	4(141)	5.28	[-5.25, 15.81]	0.33	1.10	0	
**Average training duration per session**							0.70
< 60 minutes	1(30)	8.53	[-27.07, 44.13]	N/A	N/A	N/A	

≥ 60 minutes	12(520)	1.58	[-3.10, 6.27]	0.51	6.34	0	
**Training order**							0.46
Aerobic before resistance	3(125)	5.19	[-2.21, 12.60]	0.17	1.22	0	
Resistance before aerobic	2(98)	-0.22	[-12.73, 12.29]	0.97	0.15	0	
Alternately	7(297)	-1.01	[-7.92, 5.90]	0.77	3.44	0	
**Intensity**							0.68
Moderate-intensity	4(104)	-0.68	[-13.39, 12.03]	0.92	2.90	0	
High-intensity	10(510)	2.18	[-2.67, 7.04]	0.38	3.46	0	

Meta-regression analyses were conducted to evaluate the potential moderating effects of the following continuous variables on the pooled main effect: age (β = 0.001, 95% *CI*: -0.012 to 0.014, *P* = 0.94), baseline BMI (β = -0.003, 95% *CI*: -0.036 to 0.030, *P* = 0.85), intervention duration (β = -0.004, 95% *CI*: -0.011 to 0.004, *P* = 0.31), training frequency (β = 0.013, 95% *CI*: -0.134 to 0.160, *P* = 0.86), average training duration per session (β = -0.091, 95% *CI*: -0.555 to 0.372, *P* = 0.70), and total weekly training duration (β = 0.001, 95% *CI*: -0.002 to 0.003, *P* = 0.95). The results demonstrated that none of the aforementioned continuous variables exerted a statistically significant moderating effect on the differential efficacy between CE and AE in reducing TC ($P > 0.05$).

TG

The meta-analysis, incorporating 15 independent studies, showed no statistically significant between-group difference between CE and AE in post-intervention TG levels among adolescents and adults with overweight or obesity (MD = 0.02 mmol/L, 95% CI: −0.05 to 0.09, P = 0.62). Between-study heterogeneity was low and not statistically significant (τ^2 = 0.01, I^2 = 31.87%, H^2 = 1.47; Q(14) = 17.31, P = 0.24) (Figure. 8).

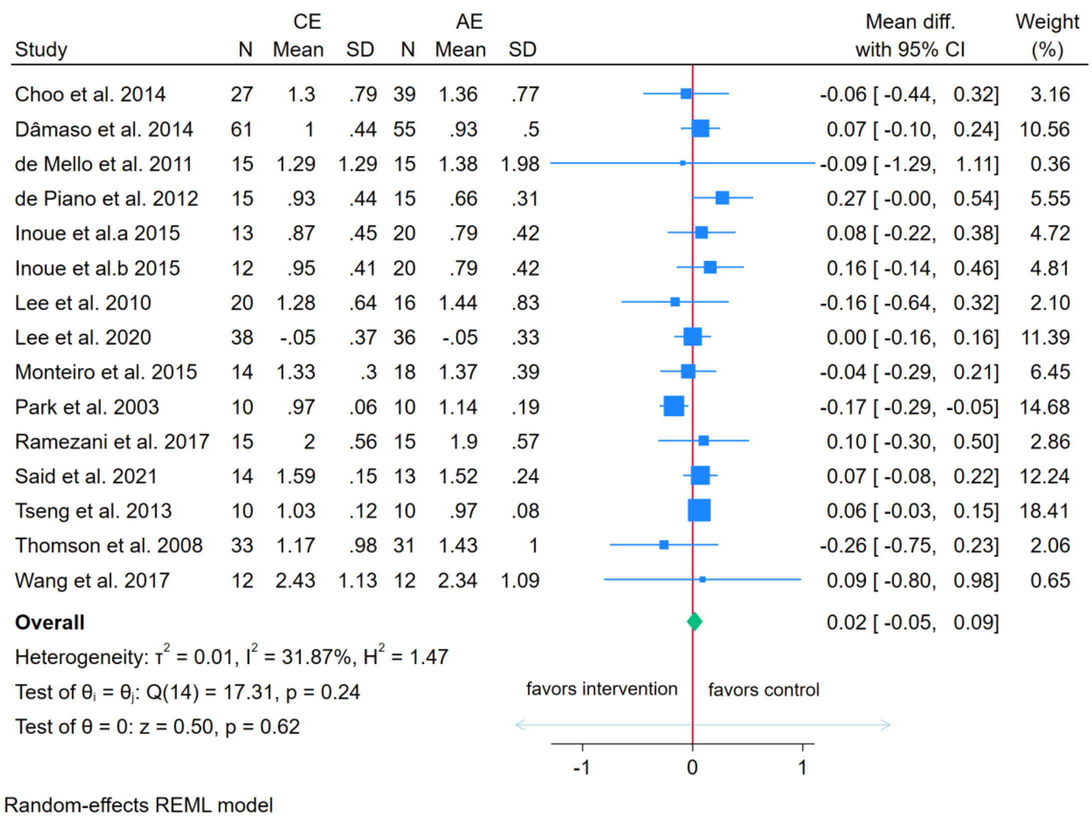

Supplemental Figure 8. Meta-analysis results for TG.

Subgroup analysis for TG (Table 13) revealed that within overweight cohorts, the efficacy of CE in reducing TG was significantly superior to that of isolated AE; conversely, within obese cohorts, the efficacy of isolated AE was superior to that of CE. Furthermore, no significant subgroup effects were observed concerning age, intervention duration, training frequency, average training duration per session, training sequence, or exercise intensity ($P > 0.05$).

Supplemental Table 13. Subgroup analysis results for TG.

Subgroup	K(n)	MD	95% CI	P_d	Q	I^2/%	P_m
**Age**							0.27
Children and adolescents	9(413)	5.02	[-2.54, 12.59]	0.19	4.89	0	
Adults	5(197)	-2.80	[-14.36, 8.76]	0.63	11.39	64	
**BMI**							<0.01
Overweight	4(146)	-13.90	[-23.82, -3.99]	0.01	0.62	0	
Obesity	11(488)	5.29	[0.20, 10.37]	0.04	5.77	0	
**Intervention period**							0.38
≤ 12 weeks	4(113)	5.33	[-1.33, 12.00]	0.12	0.89	0	
> 12 weeks	11(521)	0.31	[-8.63, 9.25]	0.95	14.27	37	
**Training frequency**							0.39

Subgroup	K(n)	MD	95% CI	P_d	Q	I^2/%	P_m
≤ 3 times/week	10(473)	4.38	[-3.12, 11.89]	0.25	5.16	0	
> 3 times/week	5(161)	-1.65	[-13.36, 10.05]	0.78	11.60	65	
**Average training duration per session**							0.71
< 60 minutes	1(30)	8.87	[-26.80, 44.54]	N/A	N/A	N/A	
≥ 60 minutes	13(540)	1.95	[-4.81, 8.71]	0.57	16.37	37	
**Training order**							0.78
Aerobic before resistance	3(125)	3.33	[-6.23, 12.88]	0.50	0.45	0	
Resistance before aerobic	2(98)	-3.91	[-22.27, 14.45]	0.68	0.01	0	
Alternately	8(317)	3.11	[-7.36, 13.59]	0.56	15.44	57	
**Intensity**							0.90
Moderate-intensity	4(104)	2.14	[-26.27, 30.54]	0.88	8.66	64	
High-intensity	11(530)	3.96	[-1.15, 9.07]	0.13	4.26	0	

Meta-regression analyses were conducted to evaluate the potential moderating effects of the following continuous variables on the pooled main effect: age (β = -0.008, 95% CI: -0.013 to -0.002, $P < 0.01$), baseline BMI (β = 0.022, 95% CI: 0.008 to 0.036, $P = 0.002$), intervention duration (β = 0.003, 95% CI: -0.002 to 0.007, P = 0.276), training frequency (β = -0.053, 95% CI: -0.112 to 0.004, $P = 0.07$), average training duration per session (β = -0.039, 95% CI: -0.257 to 0.179, $P = 0.73$), and total weekly training duration (β = -0.001, 95% CI: -0.002 to 0.001, $P = 0.08$). The results demonstrated that both age and baseline BMI exerted statistically significant moderating effects on the differential efficacy between CE and AE in reducing TG. Specifically, with advancing age, the efficacy of CE in reducing TG became significantly more pronounced compared to AE ($P < 0.01$). In contrast, higher baseline BMI was associated with a less favorable comparative effect of CE on TG, as indicated by the positive regression coefficient ($P = 0.002$), suggesting that the relative advantage of CE over AE for TG reduction diminished as baseline BMI increased (Figure. 9). Intervention duration, training frequency, average training duration per session, and total weekly training duration did not demonstrate any significant moderating effects ($P > 0.05$).

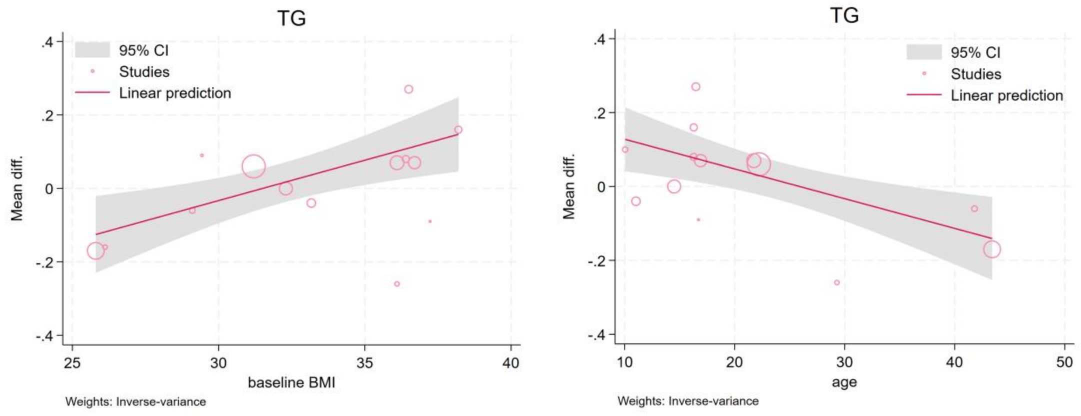

Supplemental Figure 9. Regression analysis results for TG.

LDL-C

The meta-analysis, incorporating 14 independent studies, showed no statistically significant between-group difference between CE and AE in post-intervention LDL-C levels among adolescents and adults with overweight or obesity (MD = 0.03 mmol/L, 95% CI: −0.07 to 0.13, P = 0.54). Between-study heterogeneity was negligible (τ^2 = 0.00, I^2 = 0.00%, H^2 = 1.00; Q(13) = 5.57, P = 0.96) (Figure. 10).

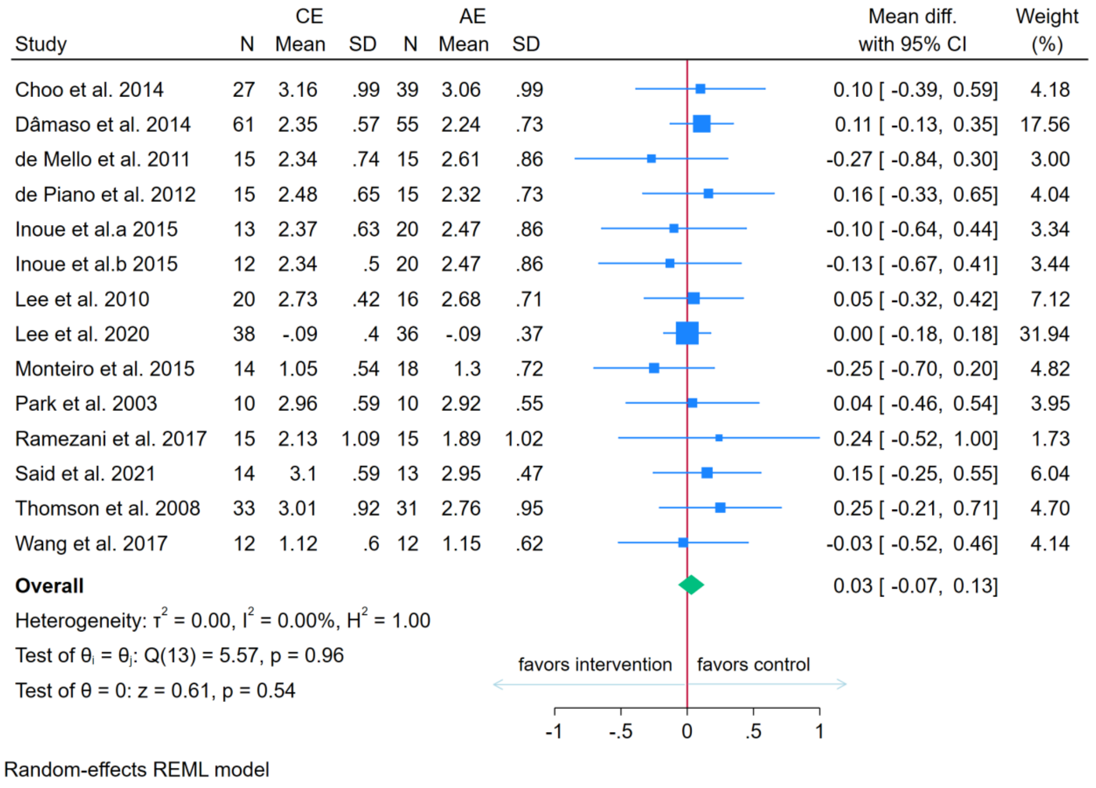

Supplemental Figure 10. Meta-analysis results for LDL-C.

Subgroup analysis for LDL-C (Table 14) revealed that no significant subgroup effects were observed concerning age, BMI classification, intervention duration, training frequency, average training duration per session, training sequence, or exercise intensity ($P > 0.05$).

Supplemental Table 14. Subgroup analysis results for LDL-C.

Subgroup	K(n)	MD	95% CI	P_d	Q	I^2/%	P_m
**Age**							0.30
Children and adolescents	9(413)	0.43	[-3.96, 4.81]	0.85	4.09	0	
Adults	4(177)	5.64	[-3.20, 14.48]	0.21	0.39	0	
**BMI**							0.93
Overweight	4(146)	1.72	[-7.00, 10.44]	0.70	0.16	0	
Obesity	10(468)	1.26	[-3.03, 5.54]	0.57	5.45	0	
**Intervention period**							0.50
≤ 12 weeks	3(93)	4.48	[-5.49, 14.45]	0.38	0.26	0	
> 12 weeks	11(521)	0.80	[-3.37, 4.97]	0.71	4.91	0	
**Training frequency**							0.25
≤ 3 times/week	10(473)	0.35	[-3.86, 4.56]	0.87	3.92	0	
> 3 times/week	4(141)	6.39	[-3.08, 15.87]	0.19	0.40	0	
**Average training duration per session**							0.57
< 60 minutes	1(30)	9.29	[-19.79,38.37]	N/A	N/A	N/A	
≥ 60 minutes	12(520)	0.78	[-3.20, 4.75]	0.70	4.40	0	
**Training order**							0.83
Aerobic before resistance	3(125)	0.98	[-4.95, 6.90]	0.75	0.49	0	
Resistance before aerobic	2(98)	-3.20	[-16.88,10.47]	0.65	1.14	13	
Alternately	7(297)	1.44	[-4.47, 7.35]	0.63	2.32	0	
**Intensity**							0.73
Moderate-intensity	4(104)	-0.26	[-10.12, 9.60]	0.96	1.27	0	
High-intensity	10(510)	1.63	[-2.54, 5.81]	0.44	4.23	0	

Meta-regression analyses were conducted to evaluate the potential moderating effects of the following continuous variables on the pooled main effect: age (β = 0.005, 95% *CI*: -0.007 to 0.018, P = 0.42), baseline BMI (β = 0.002, 95% *CI*: -0.025 to 0.030, P = 0.85), intervention duration (β = -0.001, 95% *CI*: -0.007 to 0.006, P = 0.92), training frequency (β = 0.060, 95% *CI*: -0.075 to 0.196, P = 0.38), average

training duration per session (*β* = -0.112, 95% *CI*: -0.493 to 0.269, *P* = 0.58), and total weekly training duration (*β* = 0.001, 95% *CI*: -0.002 to 0.003, *P* = 0.67). The results demonstrated that none of the aforementioned continuous variables exerted a statistically significant moderating effect on the differential efficacy between CE and AE in reducing LDL-C (*P* > 0.05).

HDL-C

The meta-analysis, incorporating 13 independent studies, showed that post-intervention HDL-C levels were modestly but significantly lower in the CE group than in the AE group (MD = −0.03 mmol/L, 95% CI: −0.06 to −0.00, P = 0.03). Because the mean difference was calculated as CE minus AE and higher HDL-C levels are generally considered favorable, this negative MD indicates a small advantage for AE rather than CE for this lipid outcome. Between-study heterogeneity was very low (τ^2 = 0.00, I^2 = 2.52%, H^2 = 1.03; Q(12) = 10.18, P = 0.60) (Figure. 11).

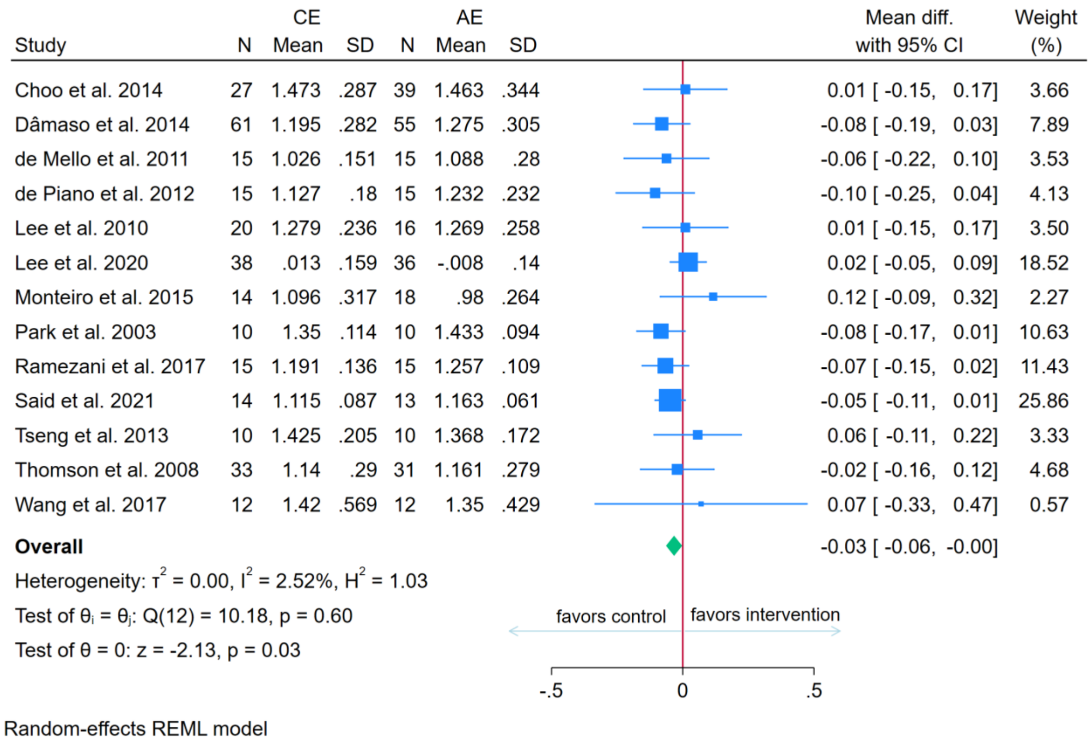

Supplemental Figure 11. Meta-analysis results for HDL-C.

Subgroup analysis for HDL-C (Table 15) revealed that no significant subgroup effects were observed concerning age, BMI classification, intervention duration,

training frequency, average training duration per session, training sequence, or exercise intensity ($P > 0.05$).

Supplemental Table 15. Subgroup analysis results for HDL-C.

Subgroup	K(n)	MD	95% CI	P_d	Q	I^2/%	P_m
**Age**							0.70
Children and adolescents	7(348)	-1.12	[-3.00, 0.76]	0.24	6.94	18	
Adults	5(197)	-1.62	[-3.26, 0.03]	0.05	2.68	0	
**BMI**							0.79
Overweight	4(146)	-1.61	[-4.37, 1.15]	0.25	1.88	2	
Obesity	9(423)	-1.20	[-2.55, 0.15]	0.08	8.18	7	
**Intervention period**							0.74
≤ 12 weeks	4(113)	-1.56	[-3.28, 0.15]	0.07	2.09	0	
> 12 weeks	9(456)	-1.14	[-2.91, 0.63]	0.21	7.87	15	
**Training frequency**							0.26
≤ 3 times/week	8(408)	-0.52	[-2.41, 1.37]	0.59	6.17	8	
> 3 times/week	5(161)	-1.93	[-3.45, -0.40]	0.01	2.39	0	
**Average training duration per session**							0.45
< 60 minutes	1(30)	-2.54	[-5.95, 0.87]	N/A	N/A	N/A	
≥ 60 minutes	11(475)	-1.12	[-2.49, 0.26]	0.11	9.54	9	
**Training order**							0.22
Aerobic before resistance	3(125)	-0.55	[-3.04, 1.94]	0.67	2.45	37	
Resistance before aerobic	2(98)	1.95	[-2.85, 6.76]	0.43	0.66	0	
Alternately	6(252)	-2.28	[-4.30, -0.26]	0.03	3.37	0	
**Intensity**							0.15
Moderate-intensity	4(104)	-3.06	[-5.73, -0.40]	0.02	0.69	0	
High-intensity	9(465)	-0.84	[-2.20, 0.52]	0.23	7.35	7	

Meta-regression analyses were conducted to evaluate the potential moderating effects of the following continuous variables on the pooled main effect: age (β = -0.001, 95% *CI*: -0.004 to 0.002, *P* = 0.51), baseline BMI (β = -0.002, 95% *CI*: -0.011 to 0.006, *P* = 0.59), intervention duration (β = -0.0001, 95% *CI*: -0.003 to 0.013, *P* = 0.44), training frequency (β = -0.02, 95% *CI*: -0.05 to 0.13, *P* = 0.25), average training duration per session (β = 0.02, 95% *CI*: -0.03 to 0.07, *P* = 0.48), and total weekly training duration (β = -0.0003, 95% *CI*: -0.0009 to 0.0002, *P* = 0.23). The results demonstrated that none of the aforementioned continuous variables exerted a statistically significant moderating effect on the between-group difference in post-intervention HDL-C levels between CE and AE ($P > 0.05$).
